# Genomic analyses of multidrug-resistant *Salmonella* Indiana, Typhimurium, and Enteritidis isolates using MinION and MiSeq sequencing technologies

**DOI:** 10.1371/journal.pone.0235641

**Published:** 2020-07-02

**Authors:** Zhao Chen, Dai Kuang, Xuebin Xu, Narjol González-Escalona, David L. Erickson, Eric Brown, Jianghong Meng

**Affiliations:** 1 Joint Institute for Food Safety and Applied Nutrition, Center for Food Safety and Security Systems, University of Maryland, College Park, Maryland, United States of Amrica; 2 Ruijin Hospital, School of Medicine, Shanghai Jiao Tong University, Shanghai, China; 3 Shanghai Municipal Center for Disease Control and Prevention, Shanghai, China; 4 Center for Food Safety and Applied Nutrition, U.S. Food and Drug Administration, College Park, Maryland, United States of America; Cornell University, UNITED STATES

## Abstract

We sequenced 25 isolates of phenotypically multidrug-resistant *Salmonella* Indiana (n = 11), Typhimurium (n = 8), and Enteritidis (n = 6) using both MinION long-read [SQK-LSK109 and flow cell (R9.4.1)] and MiSeq short-read (Nextera XT and MiSeq Reagent Kit v2) sequencing technologies to determine the advantages of each approach in terms of the characteristics of genome structure, antimicrobial resistance (AMR), virulence potential, whole-genome phylogeny, and pan-genome. The MinION reads were base-called in real-time using MinKnow 3.4.8 integrated with Guppy 3.0.7. The long-read-only assembly, Illumina-only assembly, and hybrid assembly pipelines of Unicycler 0.4.8 were used to generate the MinION, MiSeq, and hybrid assemblies, respectively. The MinION assemblies were highly contiguous compared to the MiSeq assemblies but lacked accuracy, a deficiency that was mitigated by adding the MiSeq short reads through the Unicycler hybrid assembly which corrected erroneous single nucleotide polymorphisms (SNPs). The MinION assemblies provided similar predictions of AMR and virulence potential compared to the MiSeq and hybrid assemblies, although they produced more total false negatives of AMR genotypes, primarily due to failure in identifying tetracycline resistance genes in 11 of the 19 MinION assemblies of tetracycline-resistant isolates. The MinION assemblies displayed a large genetic distance from their corresponding MiSeq and hybrid assemblies on the whole-genome phylogenetic tree, indicating that the lower read accuracy of MinION sequencing caused incorrect clustering. The pan-genome of the MinION assemblies contained significantly more accessory genes and less core genes compared to the MiSeq and hybrid assemblies, suggesting that although these assemblies were more contiguous, their sequencing errors reduced accurate genome annotations. Our research demonstrates that MinION sequencing by itself provides an efficient assessment of the genome structure, antimicrobial resistance, and virulence potential of *Salmonella*; however, it is not sufficient for whole-genome phylogenetic and pan-genome analyses. MinION in combination with MiSeq facilitated the most accurate genomic analyses.

## Introduction

Whole-genome sequencing (WGS) has been widely employed in foodborne outbreak investigations and pathogen surveillance [1]. In addition to the rapid identification of pathogens from contaminated sources of outbreaks, more detailed information about the pathogens, such as antimicrobial resistance (AMR), virulence, and inference of possible links between the sources of contamination, can also be obtained [2]. Illumina short-read sequencing technology has proven to be robust for characterizing pathogens that may have caused foodborne outbreaks and identifying those that could pose potential threats to public health [3]. However, this technology is unable to resolve repetitive and GC-rich regions, thus producing unresolvable loops in the underlying genome assembly that are fragmented into independent contigs [4]. The gaps between fragments can lead to an inability to obtain the complete whole-genome structure, which is critical in determining if some genes are co-regulated or co-transmissible and if they are located on chromosome or plasmids [5]. Moreover, the possibility of failing to identify key virulence genes during an outbreak investigation can also have negative impacts on public health assessment.

Nanopore sequencing technology that generates long reads can facilitate the completion of bacterial genome assemblies that are either lacking in sequencing depth at some repetitive regions or have areas that are missing reads completely using short-read sequencing technology [6]. Nanopore long reads can span the wide repetitive regions and also resolve GC-rich regions. Nanopore sequencing technology for full-length genome sequencing could allow the low-cost access of information necessary for making critical public health decisions.

However, Nanopore sequencing technology exhibits lower read accuracy which may produce systematic errors, and for this reason, it has previously only been applied as a complement to short-read sequencing [7]. Since the release of the MinION platform by Oxford Nanopore Technologies, nanopore chemistry, basecalling, and bioinformatic tools have been steadily evolving, with the objective of using raw Nanopore long reads independently to acquire more accurate bacterial genomes independent of other sequencing technologies [8]. In addition, closed whole-genome assemblies can also be accomplished with a combination of both short reads for base-calling accuracy and long reads for structural integrity using hybrid assembly approaches such as those found in the Unicycler and SPAdes pipelines [9, 10]. Unicycler was developed as an assembly pipeline for bacterial genomes that can conduct a hybrid assembly using both short and long reads [9]. It produces a short-read assembly graph and then uses long reads to build bridges to resolve all repeats in the genome and performs multiple rounds of short-read polishing, ultimately resulting in a complete genome assembly. The use of the Unicycler hybrid assembly with Illumina short-reads and Nanopore long reads to complete bacterial genomes has been previously reported [11–13].

*Salmonella enterica* subsp. *enterica* includes more than 2,500 different serotypes, and is considered a primary pathogen for both humans and animals worldwide [14]. The majority of the infections in humans are associated with the consumption of foods that have been contaminated by *Salmonella* [15]. By providing definitive genotypic information, WGS is ideal for investigating the emergence and dissemination of antimicrobial resistance genes (ARGs) and chromosomal point mutations that predict AMR profiles, including compounds not routinely tested phenotypically [16]. Bacteria showing identical phenotypic resistance regulated by different mechanisms can also be differentiated by WGS. An *in silico* approach to predict AMR patterns based on WGS data requires comprehensive and accurate ARG databases, as well as bioinformatic tools that can reliably detect ARGs. Here, AMRFinder (https://github.com/ncbi/amr) and the Bacterial Antimicrobial Resistance Reference Gene Database (https://www.ncbi.nlm.nih.gov/pathogens/isolates#/refgene/) are publicly available for rapid identification of AMR-related genotypes.

In this study, we sequenced 25 phenotypically multidrug-resistant isolates of *S*. Indiana, Typhimurium, and Enteritidis using both MinION and MiSeq sequencing technologies. The MinION, MiSeq, and hybrid assemblies were then compared in terms of the characteristics of genome structure, antimicrobial resistance profile, virulence potential, whole-genome phylogeny, and pan-genome. A customized, reproducible bioinformatic workflow that employs publicly available tools was developed to obtain a complete circular bacterial chromosome and its associated plasmids. These closed genomes can provide valuable information on the genome structure of *Salmonella* and complement existing characterization data from other sequencing technologies such as MiSeq. This work represents a data-driven methodology comparison through elucidating the differences, as well as similarities, between genome assemblies of bacterial foodborne pathogens obtained using MinION and MiSeq sequencing technologies.

## Materials and methods

### Bacterial strains

A total of 25 *S*. *enterica* subsp. *enterica* isolates, including serovars Indiana (n = 11), Typhimurium (n = 8), and Enteritidis (n = 6) from humans (n = 18), chicken (n = 3), seafood (n = 1), pork (n = 1), beef (n = 1), and duck (n = 1) in Shanghai, China between 2010 and 2014, were selected for this study (Table 1). All isolates were stored in tryptic soy broth (TSB; Fisher Scientific Inc., Hampton, NH) with 20% glycerol at -80°C until use.

**Table 1:** The hybrid, MinION, and MiSeq assemblies of *Salmonella* isolates.

Serotype	Isolate ID	Source	Number of contigs^a^			Total length (bp)			GC content (%)		
			Hybrid	MinION	MiSeq	Hybrid	MinION	MiSeq	Hybrid	MinION	MiSeq
Indiana	43	Seafood	1	1	114	4,993,824	4,984,753	4,936,551	51.80	51.93	51.76
	67	Chicken	2	2	105	5,072,601	5,060,699	5,020,783	51.79	51.90	51.75
	85	Chicken	4	4	132	4,991,315	5,060,699	4,932,554	51.91	51.90	51.87
	96	Human	2	2	107	4,973,668	4,962,533	4,935,343	51.86	51.98	51.81
	102	Human	6	3	99	4,985,118	4,962,779	4,922,609	51.89	52.04	51.85
	108	Human	2	2	131	5,071,476	5,061,911	4,993,042	52.01	52.14	51.97
	111	Human	5	2	146	4,899,773	4,873,314	4,826,124	52.01	52.14	52.00
	115	Human	2	2	86	4,974,921	4,957,216	4,919,970	51.85	51.98	51.81
	170	Human	2	2	110	5,028,741	5,015,229	4,971,399	51.80	51.91	51.76
	173	Pork	3	3	102	4,838,602	4,887,971	4,832,973	52.07	52.18	52.06
	174	Beef	1	1	166	4,929,169	4,918,265	4,858,029	51.95	52.06	51.93
Typhimurium	45	Human	3	4	105	5,259,999	5,287,617	5,205,240	51.94	52.07	51.90
	46	Human	3	3	125	5,259,220	5,244,083	5,206,196	51.95	52.09	51.91
	53	Human	4	4	97	5,185,065	5,120,128	5,123,654	52.15	52.28	52.12
	56	Human	6	5	141	5,210,197	5,193,224	5,123,456	52.24	52.34	52.18
	90	Duck	5	5	130	5,177,998	5,165,774	5,103,046	52.22	52.37	52.18
	101	Human	3	3	166	5,225,110	5,255,160	5,099,151	52.15	52.28	52.09
	106	Human	3	3	90	5,195,418	5,184,502	5,147,440	51.86	51.95	51.83
	113	Human	2	2	119	5,121,995	5,110,567	5,071,774	52.18	52.31	52.15
Enteritidis	74	Human	3	3	71	4,859,063	4,848,928	4,826,041	52.15	52.25	52.12
	81	Human	2	3	44	4,797,988	4,828,175	4,761,547	52.16	52.25	52.13
	95	Human	5	4	99	4,839,384	4,828,999	4,816,051	52.18	52.28	52.14
	104	Human	4	4	67	4,848,540	4,839,326	4,811,720	52.19	52.29	52.15
	109	Human	3	3	72	4,829,486	4,820,259	4,801,197	52.17	52.27	52.15
	124	Chicken	3	3	57	4,847,360	4,835,849	4,806,481	52.19	52.29	52.16

^a^All contigs of the hybrid and MinION assemblies were circularized. The hybrid and MinION assemblies that had more than one contigs contained one chromosome and one or more plasmids.

### Antimicrobial Susceptibility Testing (AST)

AST was performed using 18 antibiotics, including ampicillin (AMP), amoxicillin/clavulanic acid (AMC), ceftiofur (EFT), cephalexin (CEP), ceftriaxone (CRO), cefoxitin (FOX), chloramphenicol (CHL), nalidixic acid (NAL), ciprofloxacin (CIP), levofloxacin (LVX), ofloxacin (OFX), amikacin (AMK), gentamicin (GEN), kanamycin (KAN), streptomycin (STR), tetracycline (TET), sulfafurazole (SUL), and sulfamethoxazole/trimethoprim (SXT). Minimum inhibitory concentrations (MICs) were determined by broth microdilution using dehydrated panels CMV2AGNF and CMV3AGNF (Fisher Scientific Inc.) following standard protocols. AMR breakpoints were defined using the Clinical and Laboratory Standards Institute (CLSI) standards [17].

### Genomic DNA extraction

*Salmonella* isolates were grown in 20 ml of TSB with 0.6% yeast extract (YE; Fisher Scientific Inc.) overnight at 37°C with agitation at 150 rpm. For MiSeq sequencing, genomic DNA was extracted using the DNeasy Blood and Tissue Kit (QIAGEN Inc., Valencia, CA) on a QIAcube robotic workstation (QIAGEN Inc.). For MinION sequencing, genomic DNA was extracted using the Blood & Cell Culture DNA Maxi Kit (QIAGEN Inc.) following the manufacturer’s instructions. The mixture of bacterial cells, lysozyme, RNase A, and QIAGEN Protease was incubated at 37°C for an extended period of time (1 h) to ensure complete cell lysis, as well as complete RNA and protein degradations. DNA was precipitated by inverting the tube containing Buffer QF and isopropanol 10–20 times and spooled using an inoculating needle. The spooled DNA was immediately transferred to a microcentrifuge tube containing 0.2 ml of Tris-EDTA (TE) buffer, pH 8.0 (Fisher Scientific Inc.) and then dissolved at 55°C for 2 h. Genomic DNA was stored at 4°C until use. DNA concentrations were measured using the Qubit dsDNA HS Assay Kit (Fisher Scientific Inc.) on a Qubit 3.0 fluorometer (Fisher Scientific Inc.).

### Library preparation and WGS

MiSeq libraries were prepared with 1 ng of genomic DNA input using the Nextera XT DNA Library Preparation Kit (Illumina Inc., SanDiego, CA) following the manufacter’s instructions. Afterwards, libraries were sequenced using the MiSeq Reagent Kit v2 (500-cycles) (Illumina Inc.) on a MiSeq System using the 2x250 bp pair-end chemistry. The adapter trimming option in the Illumina FASTQ file generation pipeline was used to remove adapter sequences from the 3’ ends of the reads.

For MinION sequencing, libraries were prepared using the Ligation Sequencing Kit (Oxford Nanopore Technologies Inc., Oxford, UK) using the 1D Genomic DNA by Ligation protocol (SQK-LSK109), with a minor modification that 4 μg of genomic DNA input was added as the initial input instead of the recommended amount of 1 μg. The prepared libraries were loaded into a MinION flow cell (R9.4.1) and sequenced on the MinION device. The sequenced reads were base-called in real-time using MinKnow 3.4.8 integrated with Guppy 3.0.7. After each use, flow cells were washed using the Flow Cell Wash Kit (Oxford Nanopore Technologies Inc.) and stored at 4°C. Flow cells were reused if the available pore number were above 800 based on the quality check.

### Bioinformatic workflow

**Fig 1:**
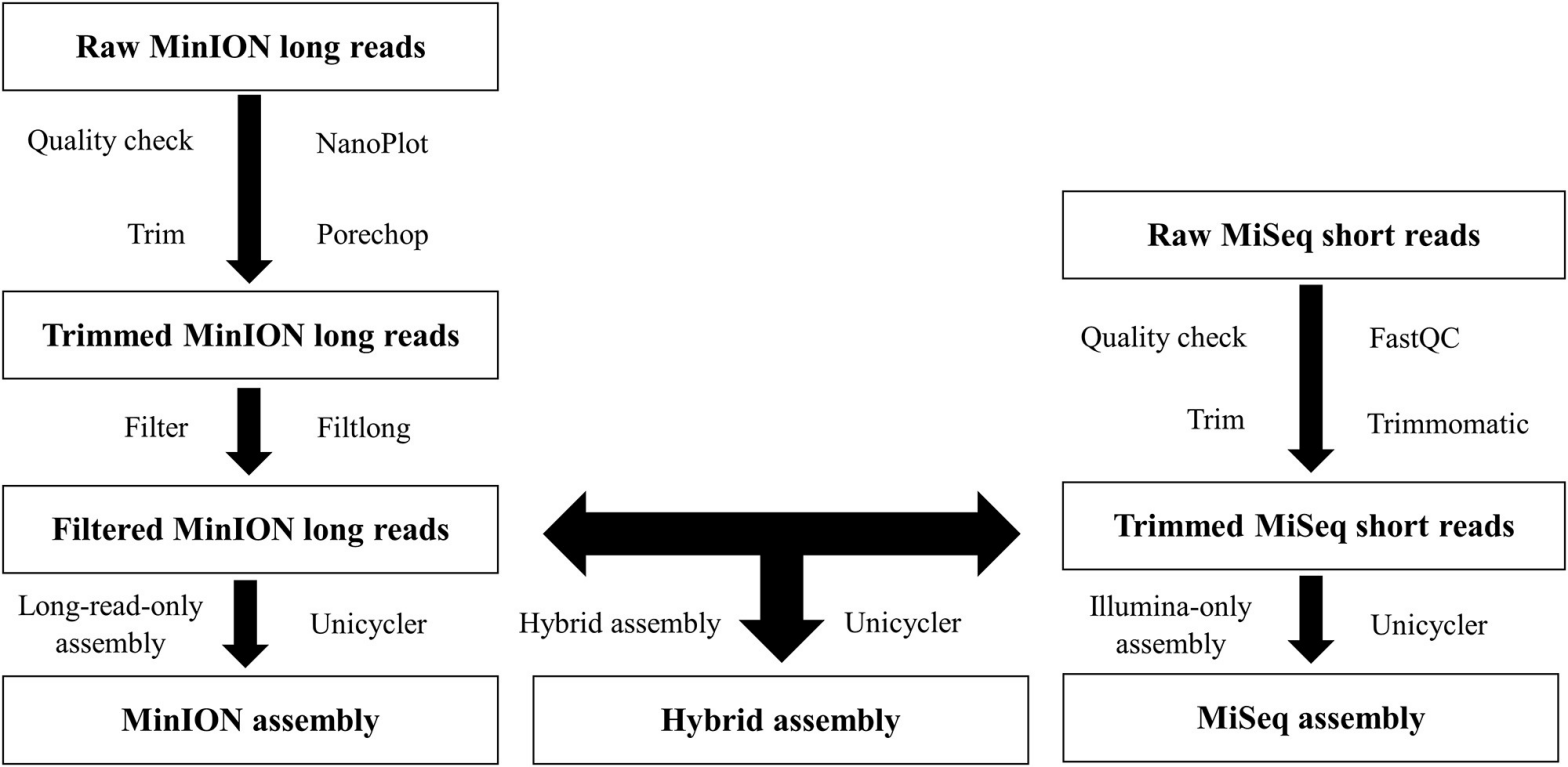
Bioinformatic workflow of the hybrid, MinION, and MiSeq assemblies.

The quality of the raw short reads of MiSeq was checked using FastQC 0.11.9 (https://github.com/s-andrews/FastQC). Q-score was used to predict the probability of an error in base-calling. Over 75% of bases >Q30 averaged across the entire run was considered acceptable for MiSeq Reagent Kit v2 (2×250 bp). The bioinformatic workflow of the hybrid, MinION, and MiSeq assemblies is shown in Fig 1. Raw reads were trimmed using Trimmomatic 0.36.4 [18], following the SLIDINGWINDOW operation with four bases to average across and 20 as the average quality required. Trimmed reads were then *de novo* assembled with the Illumina-only assembly method in the Unicycler 0.4.8 pipeline [9], which functions mainly as an optimizer of SPAdes 3.13.1 [19].

The mean read quality of the raw long reads of MinION was scored using NanoPlot 1.0.0 [20]. The adapters on the ends of the raw reads were trimmed with Porechop 0.2.4 (https://github.com/rrwick/Porechop). When a read has an adapter in its middle, it is regarded as chimeric and cleaved into two separate reads with the adapter subsequently removed. Trimmed reads were then subsampled for subsequent assembly using Filtlong 0.2.0 (https://github.com/rrwick/Filtlong). Filtlong subsampling was not random but more weight was given to read quality. The selections of minimum length and minimum window quality were relatively conservative, as this was necessary to ensure a sufficient coverage of small plasmids. All read lengths were retained and 50 was designated as the minimum window quality. The worst 10% of the MinION long reads, as measured by bases, was discarded to further increase read quality. To determine when to stop the MinION sequencing process, a long-read-only assembly of the trimmed, filtered reads was conducted with Miniasm 0.3 (https://github.com/lh3/miniasm), followed by multiple rounds of polishing with Racon 1.4.3 [21], in the Unicycler pipeline to see if sufficient data were gathered to generate a complete genome assembly. The trimmed, filtered reads were also assembled using the hybrid assembly method (normal mode) in the Unicycler pipeline, which can produce an assembly graph with the MiSeq short reads and then use the MinION long reads to build bridges to resolve all repeats. Multiple rounds of polishing were performed with the MiSeq short reads using Bowtie2 2.3.5.1 [22], Samtools 1.9 [23], and Pilon 1.23 [24] in the Unicycler pipeline to correct small errors. Finally, circularized contigs were rotated to begin at a starting gene of *dnaA* or *repA* if one could be detected with BLAST+. Bandage 0.8.1 [25] was used to visually assess the quality of *de novo* assemblies by loading the assembly graphs in GFA format after the Unicycler assembly.

The raw MinION and MiSeq reads of the 25 *Salmonella* isolates (BioSample accession numbers: SAMN14450150-SAMN14450174) were deposited into the Sequence Read Archive (SRA) database under the BioProject accession number PRJNA615288. The complete genomes based on the hybrid assemblies were submitted to the GenBank database under the accession numbers CP050706-CP050785.

### Identifications of plasmids, ARGs, chromosomal point mutations, virulence genes, and *Salmonella* pathogenicity islands (SPIs)

Plasmids were detected and typed using staramr 0.6.0 (https://github.com/phac-nml/staramr) against the PlasmidFinder database [26], and the sequences were blasted with the database to known plasmid types with 98% minimum identity and 60% minimum coverage. AMRFinder 3.0 alpha using the Bacterial Antimicrobial Resistance Reference Gene Database and staramr 0.6.0 using the PointFinder database [27] were implemented to identify ARGs and chromosomal point mutations, respectively, with 90% minimum identity and 60% minimum coverage compared with known reference sequences. Mass screening of sequences for virulence genes was performed using ABRicate 0.8.7 (https://github.com/tseemann/abricate) integrated with the Virulence Factors Database (VFDB) [28] for bacterial pathogens, with 90% minimum identity and 60% minimum coverage compared with known reference sequences. SPIFinder 1.0 (https://cge.cbs.dtu.dk/services/SPIFinder/) was used to identify SPIs with 90% minimum identity and 60% minimum coverage compared with known reference sequences.

### Whole-genome phylogenetic analysis

CSI Phylogeny 1.4 [29] was used to call single nucleotide polymorphisms (SNPs) of the MinION, MiSeq, and hybrid assemblies and then infer phylogeny based on the concatenated alignment of the high-quality SNPs. Default settings were used, with 10× as the minimum depth at SNP positions, 10% as the minimum relative depth at SNP positions, 10 bp as the minimum distance between SNPs, 30 as the minimum SNP quality, 25 as the minimum read mapping quality, and 1.96 as the minimum Z-score. *S*. Typhimurium LT2 (RefSeq assembly accession: GCF_000006945.2) served as the reference genome for SNP calling. The inferred whole-genome phylogeny in Newick format was visualized as a rectangular tree layout with Geneious Prime 2020.1.1. (Biomatters, Ltd., Auckland, New Zealand).

### Pan-genome analysis

Sequences were annotated with Prokka 1.14.0 [30] to generate annotated assemblies in GFF3 format containing both sequences and annotations for subsequent pan-genome analysis. Pan-genomes were analyzed and calculated using Roary 3.12.0 [31]. The results were visualized using the Roary plots module to generate a matrix with the presence and absence of core and accessory genes against the core-genome phylogenetic tree and a pan-genome pie chart that breaks down into the core, soft-core, shell, and cloud genes. The core-genome SNP alignment was conducted using Parsnp 1.2 [32], allowing for automatic recruitment of the reference sequence and requiring that all genomes be included for the analysis.

### Genome visualization

SnapGene 4.1.9 (GSL Biotech LLC., Chicago, IL) was used to visualize *Salmonella* genomes which were annotated with plasmids, ARGs, SPIs, clustered regularly interspaced short palindromic repeats (CRISPRs), *cas* genes, and prophage regions. CRISPRs and *cas* genes and intact prophge regions (score>90) were detected and identified using CRISPRCasFinder and PHASTER, respectively [33, 34].

### Statistical comparisons among assemblies

To evaluate if differences among the hybrid, MinION, and MiSeq assemblies were significant (*P*<0.05), the non-parametric Wilcoxon signed-rank test was used to compare values for four characters: total length, GC content, numbers of false positives of AMR genotypes, and numbers of false negatives of AMR genotypes. The test compared paired values allowing for contrast of two treatments of the hybrid, MinION, and MiSeq assemblies (MinION assembly versus MiSeq assembly, MinION assembly versus hybrid assembly, and MiSeq assembly versus hybrid assembly). All three combinations of contrast were evaluated for each of the four characters. The test was conducted in R using the Wilcoxon.test function.

## Results and discussion

### Genome assemblies

For MiSeq sequencing, more than 85% of the paired-end short reads received scores of >Q30 for each *Salmonella* isolate. For MinION sequencing, the average of the mean read length of all isolates was 20,849. The mean quality scores of the MinION long reads ranged from 10.2 to 11.2 with an average of 10.6, which corresponded to an approximate accuracy of over 90%. The *de novo* assembly using only MiSeq sequence data generated assemblies with genome sizes that ranged from 4.7 to 5.2 Mb (Table 1). Genome sizes of the MinION assemblies ranged from 4.8 to 5.2 Mb. To achieve the best possible assemblies, the hybrid assembly was carried out with the Unicycler hybrid assembly method using both short and long reads [9], which generated assemblies with genome sizes varying from 4.8 to 5.2 Mb. Total length for each of the 25 isolates was compared as paired values for three contrasts. Only the MiSeq assembly versus hybrid assembly contrast was significant (*P* = 0.02382).

**Fig 2:**
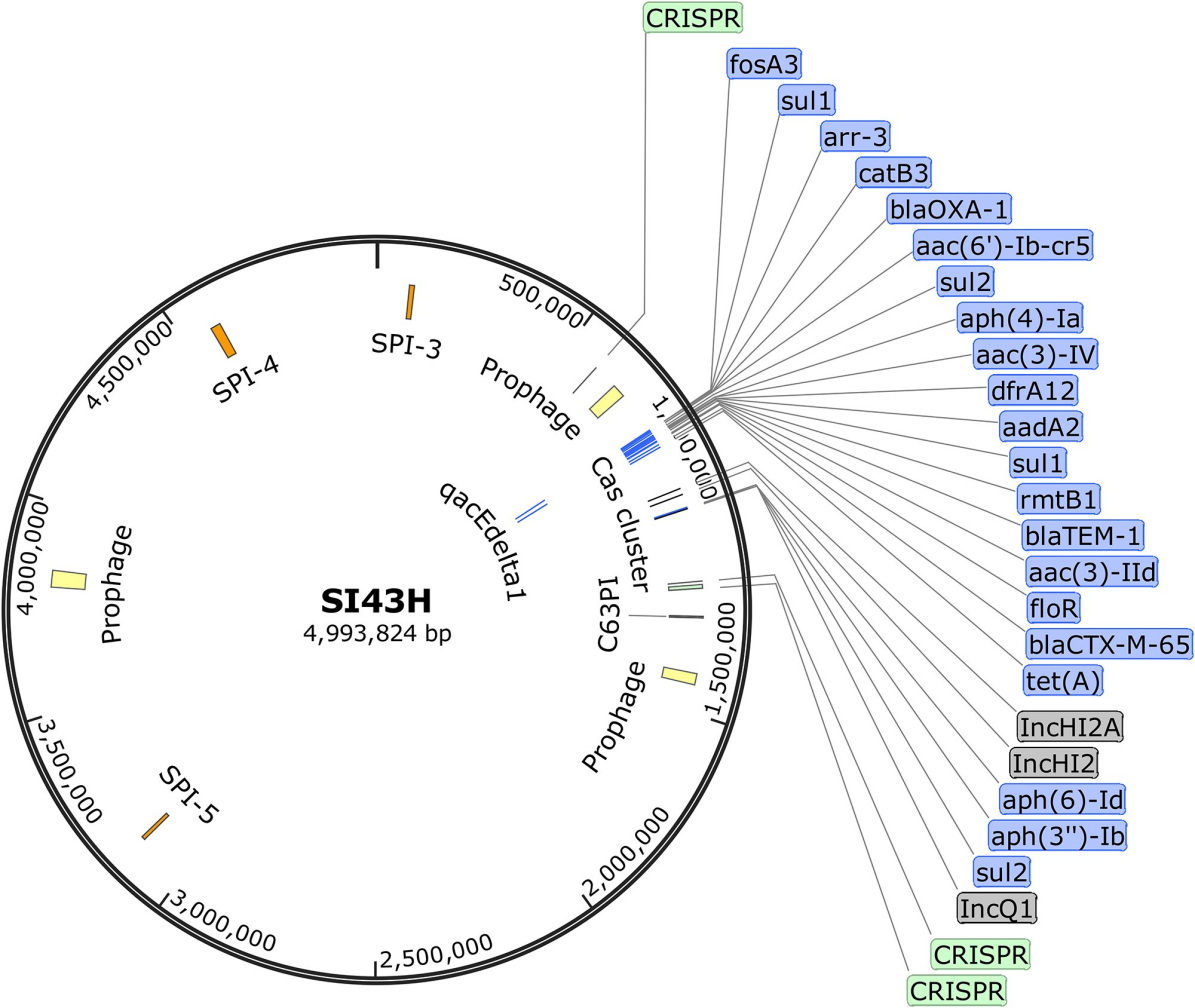
Circular chromosome map of *S*. Indiana isolate 43 (SI43H) based on its hybrid assembly.

*Salmonella* genomes were assembled into 44–166 contigs using MiSeq sequencing (Table 1). Noticeably, although there were variations in the number of contigs among the MinION assemblies, a closed bacterial genome was obtained for each isolate, including a circular chromosome and plasmid(s) (Table 1). Based on the *de novo* assembly, all isolates contained 1 to 5 plasmids except isolates 43 and 174. The sizes of the plasmids identified in those genomes ranged widely from 2 to 260 kb. The largest plasmid (260,432 bp) was detected in isolate 45. As detected by PlasmidFinder, the MinION, MiSeq, and hybrid assemblies showed consistent plasmid profiles for all isolates except isolate 102 (S1 and S2 Tables). IncX1 was detected in the MiSeq assembly of isolate 102 but not in its MinION assembly. A discrepancy was also obaserved in the number of contigs between the MinION and hybrid assemblies of isolates 102, 111, 45, 56, 81, and 95. As predicted with the MinION and hybrid assemblies, although isolate 43 had only one contig, we observed three plasmids (IncHI2, IncHI2A, and IncQ1) integrated into its chromosome (Fig 2), demonstrating an advantage of MinION in plasmid analysis over MiSeq. No plasmids were detected in isolate 174 which also had one contig. Using PlasmidFinder for plasmid analysis on the MiSeq assemblies has some major limitations. The PlasmidFinder database was developed solely based on unique short sequences (200–800 bp) of plasmid replicons for the Enterobacteriaceae family. Our data might be interpreted to suggest that this approach may not be reliable due to the fact that the entire structure of a plasmid cannot be fully revealed since recombination, insertion, or deletion events frequently occur among plasmids [35]. Therefore, it is important to acquire the full sequence of a plasmid using Nanopore sequencing technology to study its type, structure, and evolution.

Grey, plasmids; blue, antimicrobial resistance genes (ARGs); orange, *Salmonella* pathogenicity islands (SPIs); green, clustered regularly interspaced short palindromic repeats (CRISPRs) and *cas* genes; yellow, prophage regions.

Assemblies using three different methods produced GC contents of 51–52%. It should be noted that the MinION assemblies always had higher GC contents than the MiSeq assemblies, although the differences between these two methods were only 0.03–0.19%. All three contrasts were significantly different in GC content from each other (*P*<0.00001). Genome assembly using only short-read sequence data is complicated by biases that may occur during library preparation and cause some genetic regions to be excluded from the final library [36]. Common short-read library preparation methods (e.g. Illumina Nextera XT DNA Library Preparation protocol) include PCR amplification steps that are biased against regions with extreme GC contents. Library preparation methods using transposases to fragment DNA may also shear genomes, causing further biases that limit the capability of short-read sequencing [37]. Our hybrid assembly demonstrated that the MinION sequence data improved the contiguity of the MiSeq assembly when running the Unicycler hybrid assembly method (Table 1). As implemented in this mode, the MinION reads can scaffold contigs generated by short reads to build bridges over regions of the assembly graph that cannot be resolved by MiSeq sequencing alone [9]. This highlights the ability of the MinION long reads to resolve genomic repeats and reconstruct complete genomic assemblies that were otherwise fragmented when assembling using short reads only.

### ARGs and chromosomal point mutations

A number of ARGs and chromosomal point mutations were identified in the MinION, MiSeq, and hybrid assemblies, most of which were associated with antibiotics tested in AST (Tables 2–4 and S3–S5 Tables).

**Table 2:** Antimicrobial resistance (AMR) phenotypes of *S*. Indiana isolates, and their corresponding AMR genotypes, as predicted based on their hybrid, MinION, and MiSeq assemblies.

Antibiotic class^c^	Phenotype vs genotype	Method	Isolate ID										
			43	67	85	96	102	108	111	115	170	173	174
*β*-lactam	Phenotype	AST	AMP^a^, EFT, CEP, CRO	AMP, EFT, CEP, CRO	AMP, EFT, CEP, CRO	AMP	AMP, AMC, EFT, CEP, CRO	AMP, AMC, EFT, CEP, CRO	AMP, EFT, CEP, CRO	-^b^	AMP, EFT, CEP, CRO	AMP, EFT, CEP, CRO	AMP, EFT, CEP, CRO
	Genotype	Hybrid	EFT, CEP, CRO, FOX	EFT, CEP, CRO, FOX	EFT, CEP, CRO, FOX	EFT, CEP, CRO, FOX	AMP, AMC, EFT, CEP, CRO, FOX	AMP, AMC, EFT, CEP, CRO, FOX	AMP, AMC, EFT, CEP, CRO, FOX	-	EFT, CEP, CRO, FOX	AMP, AMC, EFT, CEP, CRO, FOX	EFT, CEP, CRO, FOX
		MinION	AMP, AMC, EFT, CEP, CRO, FOX	AMP, AMC, EFT, CEP, CRO, FOX	AMP, AMC, EFT, CEP, CRO, FOX	AMP, AMC, EFT, CEP, CRO, FOX	AMP, AMC, EFT, CEP, CRO, FOX	AMP, AMC, EFT, CEP, CRO, FOX	AMP, AMC, EFT, CEP, CRO, FOX	-	AMP, AMC, EFT, CEP, CRO, FOX, STR	AMP, AMC, EFT, CEP, CRO, FOX	EFT, CEP, CRO, FOX
		MiSeq	EFT, CEP, CRO, FOX	EFT, CEP, CRO, FOX	EFT, CEP, CRO, FOX	EFT, CEP, CRO, FOX	AMP, AMC, EFT, CEP, CRO, FOX	AMP, AMC, EFT, CEP, CRO, FOX	AMP, AMC, EFT, CEP, CRO, FOX	-	EFT, CEP, CRO, FOX	AMP, AMC, EFT, CEP, CRO, FOX	EFT, CEP, CRO, FOX
Aminoglycoside	Phenotype	AST	AMK, GEN, KAN, STR	GEN, KAN	AMK, GEN, KAN, STR	STR	GEN, KAN, STR	AMK, GEN, KAN, STR	AMK, GEN, KAN, STR	STR	GEN, KAN	GEN, KAN	-
	Genotype	Hybrid	AMK, GEN, KAN, STR, HYB^*e*^	AMK, GEN, STR, HYB	AMK, GEN, KAN, STR, HYB	AMK, KAN, STR	AMK, GEN, KAN, STR, HYB	AMK, GEN, KAN, STR, HYB	AMK, KAN, STR	KAN, STR	AMK, GEN, STR, HYB	AMK, KAN	AMK, GEN, KAN, STR, HYB
		MinION	AMK, GEN, KAN, STR, HYB	AMK, GEN, STR, HYB	AMK, GEN, KAN, STR, HYB	AMK, KAN, STR	AMK, GEN, KAN, STR, HYB	AMK, GEN, KAN, STR, HYB	AMK, KAN, STR	KAN, STR	AMK, GEN	AMK, GEN, KAN, STR, HYB	AMK, GEN, KAN, STR, HYB
		MiSeq	AMK, GEN, KAN, STR, HYB	AMK, GEN, STR, HYB	AMK, GEN, KAN, STR, HYB	AMK, KAN, STR	AMK, GEN, KAN, STR, HYB	AMK, GEN, KAN, STR, HYB	AMK, KAN, STR	KAN, STR	AMK, GEN, STR, HYB	AMK, GEN, KAN, STR, HYB	AMK, GEN, KAN, STR, HYB
Tetracycline	Phenotype	AST	TET	TET	-	TET	TET	TET	-	TET	TET	TET	TET
	Genotype	Hybrid	TET	TET	TET	TET	TET	TET	-	TET	TET	-	TET
		MinION	-	-	-	TET	-	-	-	TET	TET	-	TET
		MiSeq	TET	TET	TET	TET	TET	TET	-	TET	TET	TET	TET
Sulfonamide and trimethoprim	Phenotype	AST	SUL, SXT	SUL, SXT	SUL, SXT	SUL	SUL, SXT	SUL, SXT	SUL, SXT	SUL	SUL, SXT	SUL, SXT	SUL
	Genotype	Hybrid	SUL, SXT	SUL, SXT	SUL, SXT	SUL	SUL	SUL, SXT	SUL, SXT	SUL	SUL, SXT	SUL, SXT	SUL, SXT
		MinION	SUL, SXT	SUL, SXT	SUL, SXT	SUL	SUL, SXT	SUL	SUL, SXT	SUL	SUL, SXT	SUL, SXT	SUL, SXT
		MiSeq	SUL, SXT	SUL, SXT	SUL, SXT	SUL	SUL	SUL, SXT	SUL, SXT	SUL	SUL, SXT	SUL, SXT	SUL, SXT
Others not tested in AST	Genotype	Hybrid	FOS, RIF, QAC	BLM, QAC	BLM, MAC, RIF, QAC	RIF, MAC, QAC	RIF, BLM, QAC	RIF, MAC, QAC	BLM, FOS, MAC, RIF, QAC	BLM, MAC	BLM, FOS, QAC	BLM, QAC	QAC
		MinION	FOS, RIF, QAC, CST	BLM, QAC, CST	MAC, RIF, QAC, CST	RIF, MAC, QAC, CST	RIF, BLM, MAC, QAC	RIF, QAC	MAC, RIF, QAC, CST	BLM, CST	BLM, FOS, QAC, CST	BLM, QAC	QAC, CST
		MiSeq	FOS, RIF, QAC	BLM, QAC	BLM, MAC, RIF, QAC	RIF, MAC, QAC	RIF, BLM	RIF, MAC, QAC	BLM, FOS, MAC, RIF, QAC	BLM, MAC	BLM, FOS, QAC	BLM, QAC	QAC

^a^AMP, ampicillin; AMC, amoxicillin/clavulanic acid; EFT, ceftiofur; CEP, cefalexin; CRO, ceftriaxone; FOX, cefoxitin; CHL, chloramphenicol; NAL, nalidixic acid; CIP, ciprofloxacin; LVX, levofloxacin; OFX, ofloxacin; AMK, amikacin; GEN, gentamicin; KAN, kanamycin; STR, streptomycin; HYB, hygromycin; TET, tetracycline; SUL, sulfafurazole; SXT, sulfamethoxazole/trimethoprim; BLM, bleomycin; FOS, fosfomycin; MAC, macrolide; RIF, rifamycin; QAC, quaternary ammonium compounds; CST, colistin.

^b^ not detected.

^c^For the antibiotic class of phenicol, isolates 67, 85, 102, 108, 170, 173, and 174 were phenotypically resistant to CHL. For the antibiotic class of quinolone, all isolates were phenotypically resistant to NAL, CIP, LVX, and OFX. The phenotypes of the antibiotic classes of phenicol and quinolone for all isolates were consistent with their genotypes, as predicted based on their hybrid, MinION, and MiSeq assemblies.

**Table 3:** Antimicrobial resistance (AMR) phenotypes of *S*. Typhimurium isolates, and their corresponding AMR genotypes, as predicted based on their hybrid, MinION, and MiSeq assemblies.

Antibiotic class^c^	Phenotype vs genotype	Method	Isolate ID							
			45	46	53	56	90	101	106	113
*β*-lactam	Phenotype	AST	AMP^a^, EFT, CEP, CRO	AMP, EFT, CEP, CRO	AMP, EFT, CEP, CRO	AMP, AMC, EFT, CEP, CRO, FOX	AMP, AMC	AMP, EFT, CEP, CRO	AMP, EFT, CEP, CRO	AMP, EFT, CEP, CRO
	Genotype	Hybrid	AMP, AMC, EFT, CEP, CRO, FOX	AMP, AMC, EFT, CEP, CRO, FOX	EFT, CEP, CRO, FOX	EFT, CEP, CRO, FOX	AMP, AMC, EFT, CEP, CRO, FOX	AMP, AMC, EFT, CEP, CRO, FOX	EFT, CEP, CRO, FOX	AMP, AMC, EFT, CEP, CRO, FOX
		MinION	AMP, AMC, EFT, CEP, CRO, FOX	AMP, AMC, EFT, CEP, CRO, FOX	AMP, AMC, EFT, CEP, CRO, FOX	AMP, AMC, EFT, CEP, CRO, FOX	AMP, AMC, EFT, CEP, CRO, FOX	AMP, AMC, EFT, CEP, CRO, FOX	AMP, AMC, EFT, CEP, CRO, FOX	AMP, AMC, EFT, CEP, CRO, FOX
		MiSeq	AMP, AMC, EFT, CEP, CRO, FOX	AMP, AMC, EFT, CEP, CRO, FOX	EFT, CEP, CRO, FOX	EFT, CEP, CRO, FOX	AMP, AMC, EFT, CEP, CRO, FOX	AMP, AMC, EFT, CEP, CRO, FOX	EFT, CEP, CRO, FOX	AMP, AMC, EFT, CEP, CRO, FOX
Quinolone	Phenotype	AST	NAL, CIP, OFX	NAL, CIP, OFX	NAL, CIP, LVX, OFX	NAL, CIP, LVX, OFX	NAL, CIP, LVX, OFX	NAL, CIP, LVX, OFX	NAL, CIP, LVX, OFX	-^b^
Aminoglycoside	Phenotype	AST	GEN, KAN, STR	GEN, KAN, STR	GEN, KAN, STR	GEN, KAN, STR	AMK, GEN, KAN, STR	GEN, STR	KAN	GEN, STR
	Genotype	Hybrid	AMK, GEN, KAN, STR, HYB	AMK, GEN, KAN, STR, HYB	GEN, KAN, STR	AMK, GEN, KAN, STR	AMK, GEN, KAN, STR	AMK, GEN, KAN, STR	AMK, KAN, STR	GEN, KAN, STR
		MinION	AMK, KAN, STR, HYB	AMK, GEN, KAN, STR, HYB	KAN, STR	AMK, GEN, KAN, STR	GEN, STR	KAN, STR	AMK, KAN, STR	GEN, KAN, STR
		MiSeq	AMK, GEN, KAN, STR, HYB	AMK, GEN, KAN, STR, HYB	GEN, KAN, STR	AMK, GEN, KAN, STR	AMK, GEN, KAN, STR	AMK, GEN, KAN, STR	AMK, KAN, STR	GEN, KAN, STR
Tetracycline	Phenotype	AST	TET	TET	TET	TET	TET	TET	TET	TET
	Genotype	Hybrid	TET	TET	TET	TET	TET	TET	TET	TET
		MinION	-	TET	TET	-	-	TET	-	-
		MiSeq	TET	TET	TET	TET	TET	TET	TET	TET
Sulfonamide and trimethoprim	Phenotype	AST	SUL, SXT	SUL, SXT	SUL, SXT	SUL, SXT	SUL, SXT	SUL	SUL, SXT	SUL
	Genotype	Hybrid	SUL, SXT	SUL, SXT	SUL, SXT	SUL, SXT	SUL, SXT	SUL	SUL, SXT	SUL
		MinION	SUL, SXT	SUL, SXT	SUL, SXT	SUL, SXT	SUL, SXT	SUL	SUL, SXT	SUL
		MiSeq	SUL, SXT	SUL, SXT	SUL, SXT	SUL, SXT	SUL, SXT	SUL	SUL, SXT	SUL
Others not tested in AST	Genotype	Hybrid	BLM, RIF, QAC	BLM, RIF, QAC	BLM, QAC	MAC, QAC	MAC, QAC	CST	BLM, RIF, QAC	CST
		MinION	BLM, RIF, QAC, CST	BLM, QAC, CST	QAC	MAC, QAC	QAC	CST	BLM, QAC	CST
		MiSeq	BLM, RIF, QAC	BLM, RIF, QAC	BLM, QAC	MAC, QAC	MAC, QAC	CST	BLM, RIF, QAC	CST

^a^AMP, ampicillin; AMC, amoxicillin/clavulanic acid; EFT, ceftiofur; CEP, cefalexin; CRO, ceftriaxone; FOX, cefoxitin; CHL, chloramphenicol; NAL, nalidixic acid; CIP, ciprofloxacin; LVX, levofloxacin; OFX, ofloxacin; AMK, amikacin; GEN, gentamicin; KAN, kanamycin; STR, streptomycin; HYB, hygromycin; TET, tetracycline; SUL, sulfafurazole; SXT, sulfamethoxazole/trimethoprim; BLM, bleomycin; FOS, fosfomycin; MAC, macrolide; RIF, rifamycin; QAC, quaternary ammonium compounds; CST, colistin.

^b^-, not detected.

^c^For the antibiotic class of phenicol, all isolates were phenotypically resistant to CHL, which was consistent with their genotypes, as predicted based on their hybrid, MinION, and MiSeq assemblies. For the antibiotic class of quinolone, all isolates were genotypically resistant to NAL, CIP, LVX, and OFX.

**Table 4:** Antimicrobial resistance (AMR) phenotypes of *S*. Enteritidis isolates, and their corresponding AMR genotypes, as predicted based on their hybrid, MinION, and MiSeq assemblies.

Antibiotic class^c^	Phenotype vs genotype	Method	Isolate ID					
			74	81	95	104	109	124
*β*-lactam	Phenotype	AST	AMP^a^, EFT, CEP, CRO	AMP, EFT, CEP, CRO	AMP, EFT, CEP, CRO	AMP, EFT, CEP, CRO	AMP, EFT, CEP, CRO	AMP, EFT, CEP, CRO
	Genotype	Hybrid	AMP, AMC, EFT, CEP, CRO, FOX	EFT, CEP, CRO, FOX	AMP, AMC, EFT, CEP, CRO, FOX	EFT, CEP, CRO, FOX	EFT, CEP, CRO, FOX	AMP, AMC, EFT, CEP, CRO, FOX
		MinION	AMP, AMC, EFT, CEP, CRO, FOX	EFT, CEP, CRO, FOX	AMP, AMC, EFT, CEP, CRO, FOX	EFT, CEP, CRO, FOX	AMP, AMC, EFT, CEP, CRO, FOX	EFT, CEP, CRO, FOX
		MiSeq	AMP, AMC, EFT, CEP, CRO, FOX	EFT, CEP, CRO, FOX	AMP, AMC, EFT, CEP, CRO, FOX	EFT, CEP, CRO, FOX	EFT, CEP, CRO, FOX	AMP, AMC, EFT, CEP, CRO, FOX
Phenicol	Phenotype	AST	-^b^	-	-	-	-	CHL
	Genotype	Hybrid	CHL	-	CHL	CHL	CHL	CHL
		MinION	-	-	CHL	CHL	CHL	CHL
		MiSeq	CHL	-	CHL	CHL	CHL	CHL
Quinolone	Phenotype	AST	NAL	NAL	NAL	NAL	NAL	NAL
Aminoglycoside	Phenotype	AST	KAN	-	GEN, KAN	KAN, STR	KAN	KAN, STR
	Genotype	Hybrid	KAN	-	GEN, KAN	KAN, STR	KAN	KAN, STR
		MinION	KAN	-	GEN, KAN	KAN, STR	KAN	KAN, STR
		MiSeq	KAN	-	GEN, KAN	KAN, STR	KAN	KAN, STR
Tetracycline	Phenotype	AST	-	-	-	TET	-	TET
	Genotype	Hybrid	-	-	-	TET	-	TET
		MinION	-	-	-	-	-	TET
		MiSeq	-	-	-	TET	-	TET
Others not tested in AST	Genotype	Hybrid	BLM	-	BLM	BLM, FOS	BLM	BLM
		MinION	BLM	-	BLM	BLM, FOS, CST	CST	-
		MiSeq	BLM	-	BLM	BLM, FOS	BLM	BLM

^a^AMP, ampicillin; AMC, amoxicillin/clavulanic acid; EFT, ceftiofur; CEP, cefalexin; CRO, ceftriaxone; FOX, cefoxitin; CHL, chloramphenicol; NAL, nalidixic acid; CIP, ciprofloxacin; LVX, levofloxacin; OFX, ofloxacin; AMK, amikacin; GEN, gentamicin; KAN, kanamycin; STR, streptomycin; HYB, hygromycin; TET, tetracycline; SUL, sulfafurazole; SXT, sulfamethoxazole/trimethoprim; BLM, bleomycin; FOS, fosfomycin; MAC, macrolide; RIF, rifamycin; QAC, quaternary ammonium compounds; CST, colistin.

^b^-, not detected.

^c^For the antibiotic class of quinolone, all isolates were genotypically resistant to NAL, CIP, LVX, and OFX. For the antibiotic class of sulfonamide and trimethoprim, isolates 104 and 124 were phenotypically resistant to SUL, which was consistent with their genotypes, as predicted based on their hybrid, MinION, and MiSeq assemblies.

#### *β*-lactam resistance

Genes responsible for *β*-lactam resistance present in the MinION, MiSeq, and hybrid assemblies belonged to the gene families of *blaCTX-M*, *blaOXA*, *blaTEM*, and *blaCMY*. Five *β*-lactam resistance genes were unique to the MiSeq and hybrid assemblies, including *blaCTX-M-14*, *blaCTX-M-27*, *blaCTX-M-55*, *blaOXA-1*, and *blaCMY-2*, while *blaOXA* and *blaCMY* were unique to the MinION assemblies.

#### Phenicol resistance

All of the chloramphenicol-resistant isolates contained at least one phenicol resistance gene. Among chloramphenicol-resistant isolates, eight phenicol resistance genes were identified in the MiSeq assemblies, including *catB3* (63%), *cmlA1* (38%), *floR* (88%), *catA1* (19%), *catA2* (25%), *oqxA* (25%), *oqxA2* (13%), and *oqxB* (38%). Phenicol resistance genes present in the MinION assemblies of chloramphenicol-resistant isolates included *catB3* (50%), *cmlA1* (31%), *floR* (69%), *catA1* (19%), *catA2* (25%), and *oqxA* (19%). The hybrid assemblies of chloramphenicol-resistant isolates carried *catB3* (63%), *cmlA1* (31%), *floR* (81%), *catA1* (19%), *catA2* (25%), *oqxA* (31%), *oqxA2* (31%), and *oqxB* (63%).

#### Quinolone resistance

Quinolone resistance is typically conferred by chromosomal point mutations of the quinolone resistance-determining regions (QRDRs) of *gyrA*, *gyrB*, *parC*, and *parE* [38] and/or the acquisition of plasmid-mediated quinolone resistance (PMQR) genes [39]. The MinION, MiSeq, and hybrid assemblies of quinolone-resistant *Salmonella* isolates carried either the QRDR mutations or the PMQR genes. There was a high level of concordance in quinolone genotypes among the MinION, MiSeq, and hybrid assemblies of *S*. Indiana isolates, as both the *gyrA* and *parC* mutations were present, with only one exception that the *gyrA* mutation was not detected in the MinION assembly of isolate 115. We observed that the *gyrA* mutation was absent only in one MiSeq and one hybrid assembly of quinolone-resistant isolates, while five MinION assemblies of quinolone-resistant isolates did not possess the *gyrA* mutation. The *parC* mutation was present in 58% of the MiSeq assemblies and 63% of the hybrid assemblies of quinolone-resistant isolates. It is worth noting that as high as 85% of the MinION assemblies of quinolone-resistant isolates contained the *parC* mutation, suggesting that MinION was more effective in detecting the *parC* mutation.

Various PMQR genes were present in genome assemblies of quinolone-resistant isolates. A total of seven PMQR genes were identified in the MiSeq assemblies, including *aac(6')-Ib-cr5* (54%), *oqxA* (17%), *oqxA2* (25%), *oqxB* (42%), *qepA1* (4%), *qepA* (4%), and *qnrS1* (8%). In contrast, MinION was obviously not effective in identifying the PMQR genes, as only three genes [*aac(6')-Ib-cr* (54%), *oqxA* (13%), and *qnrS* (8%)] were detected. The hybrid assemblies contained *aac(6')-Ib-cr5* (54%), *oqxA* (21%), *oqxA2* (21%), *oqxB* (42%), *qepA1* (8%), and *qnrS1* (8%). We observed disagreement between phenotypes and genotypes of quinolone-resistant isolates. For isolate 113, the MinION assembly contained *qnrS*, while *qnrS1* was present in both the MiSeq and hybrid assemblies; however, this isolate was not phenotypically resistant to any quinolone antibiotics tested in this study, which could be attributed to the absence of chromosomal point mutations of the QRDRs. It has been reported that only low-level quinolone resistance could be conferred by *qnr*, whose primary contribution lies in its ability to facilitate and supplement the development of chromosomal point mutations of the QRDRs [40]. And only through interactions with chromosomal point mutations of the QRDRs could *qnr* potentially boost quinolone resistance [41–43].

#### Aminoglycoside resistance

Several distinct aminoglycoside resistance genes were detected in genome assemblies of aminoglycoside-resistant isolates. Aminoglycoside resistance genes unique to the MinION assemblies included *aac(6')-Ib-cr*, *aac(3)-II*, and *aac(3)-Iva*, whereas *aac(6')-Ib-cr5*, *aac(3)-IId*, and *rmtB1* were present only in the MiSeq and hybrid assemblies.

#### Tetracycline resistance

Phenotypic resistance to tetracycline correlated highly with the presence of known resistance determinants predicted by the MiSeq assemblies. Of the 46 *tet* genes described to date [44], three were identified in the MiSeq assemblies of tetracycline-resistant isolates, with the highest prevalence being tetracycline efflux transporter encoded by *tet(A)* (79%) and *tet(B)* (32%). Relatively rare were ribosomal protection mechanisms conferred by *tet(M)* (16%), which encodes a tetracycline-degrading enzyme. According to the genotypic predictions by the MinION assemblies, *tet(A)* and *tet(B)* were only present in seven and one tetracycline-resistant isolates, respectively. In all but one case, genotypic predictions by the hybrid assemblies for tetracycline resistance were consistent with phenotypic susceptibility data. Only one isolate (isolate 85) whose MiSeq and hybrid assemblies both carried *tet(A)* was not phenotypically resistant to tetracycline.

#### Sulfonamide and trimethoprim resistance

As detected in the MiSeq assemblies, sulfafurazole resistance was predominantly encoded by *sul1* and *sul2*, which were present in 76% and 90% of sulfafurazole-resistant isolates, respectively, with 29% of these isolates containing *sul3*. The prevalence of *sul1*, *sul2*, and *sul3* in the MinION assemblies of sulfafurazole-resistant isolates was 76%, 62%, and 10%, respectively, while these three genes were identified in 76%, 86%, and 24% of the hybrid assemblies of sulfafurazole-resistant isolates, respectively.

Among the detected genes responsible for synthesizing trimethoprim-resistant dihydrofolate reductase, *dfrA12* and *dfrA7* were present in 57% and 29% of the MiSeq assemblies of trimethoprim-resistant isolates, respectively, while they were detected in 64% and 36% of the MinION assemblies of these isolates, respectively. These two genes were identified in 57% and 36% of the hybrid assemblies of trimethoprim-resistant isolates, respectively.

#### Correlations between AMR phenotype and genotype

Theoretically, any phenotypic feature of a microorganism can be derived from its genome sequence. However, both false positives (phenotypically susceptible, genotypically resistant) and false negatives (phenotypically resistant, genotypically susceptible) of AMR genotyping may occasionally occur and have some adverse consequences [16]. In the present study, we observed instances of false positives for the MinION, MiSeq, and hybrid assemblies, indicating the presence of AMR determinants even if the phenotypic susceptibilities were below the MICs (Table 5). For example, although the MinION, MiSeq, and hybrid assemblies of isolate 96 all harbored amikacin and kanamycin resistance genes, it was not phenotypically resistant to amikacin or kanamycin. Noticeably, the MinION assemblies had similar false positives compared to the MiSeq and hybrid assemblies, although amoxicillin/clavulanic acid resistance genes were present in 16 of the 21 MinION assemblies of amoxicillin/clavulanic acid-sensitive isolates. No significant differences in the numbers of false positives of AMR genotypes were observed between the MinION, MiSeq, and hybrid assemblies (*P*>0.05).

**Table 5:** Numbers of false positives and false negatives of antimicrobial resistance (AMR) genotypes of *Salmonella* isolates for each antibiotic, as predicted based on their hybrid, MinION, and MiSeq assemblies.

Antibiotic class	Antibiotic	False positives			False negatives		
		Hybrid	MinION	MiSeq	Hybrid	MinION	MiSeq
*β*-lactam	AMP^a^	0	0	0	12	4	12
	AMC	9	16	7	1	0	0
	EFT	2	2	2	0	0	0
	CEP	2	2	2	0	0	0
	CRO	2	2	2	0	0	0
	FOX	23	23	23	0	0	0
Phenicol	CHL	4	3	4	0	0	0
Quinolone	NAL	0	0	0	0	1	0
	CIP	7	6	7	0	1	0
	LVX	9	8	9	0	1	0
	OFX	7	6	7	0	1	0
Aminoglycoside	AMK	11	10	11	0	0	0
	GEN	1	1	1	2	4	1
	KAN	5	5	5	2	3	2
	STR	4	4	5	0	0	0
Tetracycline	TET	1	0	1	1	11	0
Sulfonamide and trimethoprim	SUL	0	0	0	0	0	0
	SXT	2	2	2	0	0	0
Total		89	90	88	18	26	15

^a^AMP, ampicillin; AMC, amoxicillin/clavulanic acid; EFT, ceftiofur; CEP, cefalexin; CRO, ceftriaxone; FOX, cefoxitin; CHL, chloramphenicol; NAL, nalidixic acid; CIP, ciprofloxacin; LVX, levofloxacin; OFX, ofloxacin; AMK, amikacin; GEN, gentamicin; KAN, kanamycin; STR, streptomycin; TET, tetracycline; SUL, sulfafurazole; SXT, sulfamethoxazole/trimethoprim.

False negatives were also observed for the MinION, MiSeq, and hybrid assemblies (Table 5), implying that some isolates phenotypically resistant to certain antibiotics were annotated as genotypically susceptible. No significant differences in the numbers of false negatives of AMR genotypes were observed between the MinION, MiSeq, and hybrid assemblies (*P*>0.05). For 24 ampicillin-resistant isolates, the corresponding resistance genes were present only in 20 MinION assemblies. And these genes were absent in up to 12 MiSeq and 12 hybrid assemblies of ampicillin-resistant isolates, suggesting that they were not effective in detecting genes associated with ampicillin. For five isolates resistant to amikacin, its corresponding resistance genes were not identified in one MinION assembly, while MiSeq or hybrid successfully detected amikacin resistance genes. Genes related to kanamycin resistance were not detected in three MinION assemblies, two MiSeq assemblies, and two hybrid assemblies of 19 kanamycin-resistant isolates. For 16 gentamicin-resistant isolates, the corresponding resistance genes were absent in four MinION assemblies, one MiSeq assembly, two hybrid assemblies. Nineteen isolates were observed to display phenotypic resistance to tetracycline. Tetracycline resistance genes were not identified in 11 of the 19 MinION assemblies of tetracycline-resistant isolates. In contrast, *tet* genes were identified in all MiSeq assemblies of these isolates, while they were absent only in one hybrid assembly.

Among large-scale studies investigating the correlation between phenotypes and genotypes, Feldgarden et al. [45] examined the consistency between AMR genotypes predicted using AMRFinder and resistance phenotypes of 5,425 *Salmonella* isolates from the National Antimicrobial Resistance Monitoring System. They indicated that overall, the presence or absence of kanamycin and gentamicin resistance genes was a good predictor of phenotypic susceptibility. Nonetheless, 67 out of 3,883 isolates (2%) that carried no kanamycin resistance genes still displayed phenotypic resistance to kanamycin. Similarly, 1% of isolates (53/5,419) were phenotypically resistant to gentamicin regardless of the absence of corresponding resistance genes. Other studies on *Salmonella* have also demonstrated that phenotypic breakpoints do not always correspond to the presence or absence of ARGs [46, 47]. Tyson et al. [47] also reported 10 out of 1,028 *Salmonella* isolates (1%) devoid of tetracycline resistance genes were still phenotypically resistant to tetracycline. These false negatives require closer attention, which may result in inadequate treatment of infections by resistant strains. It is generally preferable to minimize false negatives at the expense of increasing the false-positive rate, although false positives can lead to antibiotic misuse, potentially increasing the risk of resistance to last-line antibiotics.

Overall, the MinION assemblies provided similar predictions of AMR compared to the MiSeq and hybrid assemblies, although they created more total false negatives of AMR genotypes mainly due to no identified tetracycline resistance genes in 11 of the 19 MinION assemblies of tetracycline-resistant isolates.

#### Correlations between AMR and CRISPRs, cas genes, and prophage regions

Bacteria are able to meet the evolutionary challenge of combating antimicrobial chemotherapy, often by acquiring preexisting AMR determinants from the bacterial gene pool through the concerted activities of mobile genetic elements, including insertion sequences, transposons, gene cassettes/integrons, plasmids, and integrative conjugative elements [48]. Together, these elements can facilitate horizontal genetic exchange, therefore promoting the acquisition and spread of ARGs. The Bacterial Antimicrobial Resistance Reference Gene Database used in this study contains the total complement of known ARGs, not just those in *Salmonella*. Meanwhile, this approach permits the identification of ARGs that are able to cross species and ecological barriers. Interestingly, for isolate 43 with 3 plasmids integrated into the chromosome, its ARG-rich region was flanked by several CRISPRs, *cas* genes, and prophage regions (Fig 2). Multiple reports have demonstrated that CRISPR-Cas systems may play a major role in controlling horizontal gene transfer through mobile genetic elements such as plasmids and bacteriophages, which consequently protect the dynamics of ARG acquisition in bacteria [49]. DiMarzio et al. [50] observed an association between CRISPR-multi-virulence-locus sequence typing and AMR in *S*. Typhimurium isolates, but exceptions also existed under some conditions. Previous studies have also demonstrated that antibiotics such as carbadox and fluoroquinolones induced prophages that were integrated into the chromosome of *S*. Typhimurium, and also facilitated horizontal gene transfer from multidrug-resistant *S*. Typhimurium to a susceptible bacterial host strain [51, 52]. With the advent of Nanopore sequencing technology which enables the sequencing and assembly of complete bacterial genomes, it is becoming increasingly feasible to further explore the correlations between AMR and CRISPRs, *cas* genes, and mobile genetic elements. Nanopore long reads enable the characterization of mobile genetic elements on which key AMR determinants are located and also identify the combination of different AMR determinants co-located on the same mobile genetic element.

Despite some remaining limitations, the information provided by MinION and its combination with MiSeq will largely enhance the monitoring of ARGs circulating among humans, animals, foods, and environments. Nanopore sequencing technology has particular potential for rapid AMR genotyping because sequence data become readily available within minutes of starting the sequencing run. Previous studies have proved the “streaming” genome-based prediction of bacterial AMR phenotype using MinION, where the AMR profile was acquired in real-time as the sequence data were produced by the device [53, 54]. Correspondingly, MinION can be helpful to identify emerging AMR hazards more quickly and implement timely control strategies designed to mitigate potential risks to public health.

### Virulence genes and SPIs

In most cases, virulence genes in the MinION, MiSeq, and hybrid assemblies of each isolate were consistent (Table 6). The MinION assemblies included all virulence genes that were present in the MiSeq and hybrid assemblies, confirming that MinION can facilitate a rapid and accurate assessment of virulence potential by detecting specific virulence genes. Most notably, the MinION assembly of isolate 53 harbored the *rck* gene (94.1% coverage, 100% identity) responsible for resistance to complement killing, whereas this gene was absent in the corresponding MiSeq assembly. The *rck* gene was also detected in its hybrid assembly, demonstrating the utility of the MinION long reads when running the Unicycler hybrid assembly method. Similarly, when González-Escalona et al. [55] compared MiSeq, MinION, and PacBio assemblies of three clinical and environmental isolates of Shiga toxin-producing *Escherichia coli* (STEC), the MinION assemblies provided sufficient data to cover all virulence genes, which were consistent with the data of PacBio assemblies. However, several virulence genes were not detected in some MiSeq assemblies, pointing to the library preparation as being responsible for the loss of these genetic regions. Interestingly, we noticed the *shdA* gene involved in synthesizing AIDA autotransporter-like protein was identified in both MinION (99.0% coverage, 96.0% identity) and MiSeq (63.1% coverage, 94.6% identity) assemblies of isolate 95, but absent in its hybrid assembly. This discrepancy could be attributed to the frameshifts introduced during the Unicycler hybrid assembly.

**Table 6:** Numbers of virulence genes and *Salmonella* pathogenicity islands (SPIs) of *Salmonella* isolates, as predicted based on their hybrid, MinION, and MiSeq assemblies.

Serotype	Isolate ID	Number of virulence genes			Number of SPIs		
		Hybrid	MinION	MiSeq	Hybrid	MinION	MiSeq
Indiana	43	91	91	91	4	4	5 (SPI-1)
	67	92	92	92	5	5	5
	85	91	91	91	5 (SPI-4)	5 (SPI-4)	4
	96	91	91	91	5 (SPI-3)	5 (SPI-3)	5 (SPI-1)
	102	91	91	91	6	6	6
	108	91	91	91	5	6 (SPI-1)	5
	111	91	91	91	4	4	5 (SPI-1)
	115	91	91	91	5	5	6 (SPI-1)
	170	92	92	92	5	6 (SPI-1)	6 (SPI-1)
	173	88	88	88	4	4	4
	174	93	93	93	5	6 (SPI-1)	6 (SPI-1)
Typhimurium	45	103	103	103	11 (SPI-1, SPI-4)	11 (SPI-4)	10 (SPI-1)
	46	103	103	103	11 (SPI-1, SPI-4)	11 (SPI-4)	10 (SPI-1)
	53	118 (*rck*)^a^	118 (*rck*)	117	11 (SPI-1, SPI-4)	11 (SPI-4)	10 (SPI-1)
	56	118	118	118	11 (SPI-1, SPI-4)	11 (SPI-4)	10 (SPI-1)
	90	116	116	116	11 (SPI-1, SPI-4)	11 (SPI-4)	10 (SPI-1)
	101	103	104	103	12 (SPI-4)	12 (SPI-4)	11
	106	103	103	103	11 (SPI-1, SPI-4)	11	10 (SPI-1)
	113	103	103	103	11 (SPI-4)	12 (SPI-4)	10
Enteritidis	74	108	108	108	11 (SPI-12)	11 (SPI-12)	10
	81	108	108	108	11 (SPI-12)	11 (SPI-12)	10
	95	107	108 (*shdA*)	108 (*shdA*)	11 (SPI-12)	11 (SPI-12)	10
	104	108	108	108	11 (SPI-4, SPI-12)	11 (SPI-4, SPI-12)	9
	109	108	108	108	11 (SPI-12)	11 (SPI-12)	10
	124	108	108	108	11 (SPI-12)	11 (SPI-12)	10

^a^Virulence genes and SPIs in the parentheses indicate they were detected only by one or two methods.

Overall, the MinION, MiSeq, and hybrid assemblies possessed similar profiles of SPIs for each isolate (Table 5 and S6 Table). Nevertheless, some discrepancies were observed between the MinION and MiSeq assemblies. SPI-4 and SPI-12 were absent in the MiSeq assemblies of *S*. Typhimurium and Enteritidis, respectively, but identified in most MinION assemblies. These SPIs may be located at particularly repetitive or bias-prone regions such that they were omitted from the MiSeq assemblies, while they were present in the MinION assemblies that are less sensitive to these issues. SPI-1 was absent in some MinION assemblies but was detected in the MiSeq assemblies. The Unicycler hybrid assembly took into consideration both long-read and short-read data. For example, SPI-4 and SPI-1 were identified in the MinION and MiSeq assemblies respectively for isolate 45, whereas both of these SPIs were present in its hybrid assembly. Although SPI-1 was present in either the MinION or MiSeq assemblies of several *S*. Indiana isolates, it was absent in their hybrid assemblies. For isolate 106, SPI-4 was not identified in either the MinION or MiSeq assembly but was present in its hybrid assembly. Technological improvements in genome assemblers are therefore necessary to ensure raw sequence data are correctly assembled. Furthermore, enhanced genome assembly is also essential to fully understand and exploit complex genomic features such as SPIs. As more WGS data of *Salmonella* become publicly available, genomic analysis can provide a more comprehensive insight into the distribution, diversity, and host specificity of SPIs. Such information not only allows us to identify highly virulent strains and serotypes but also helps us to understand the evolution of *Salmonella* pathogenicity.

### Whole-genome phylogenetic analysis

**Fig 3:**
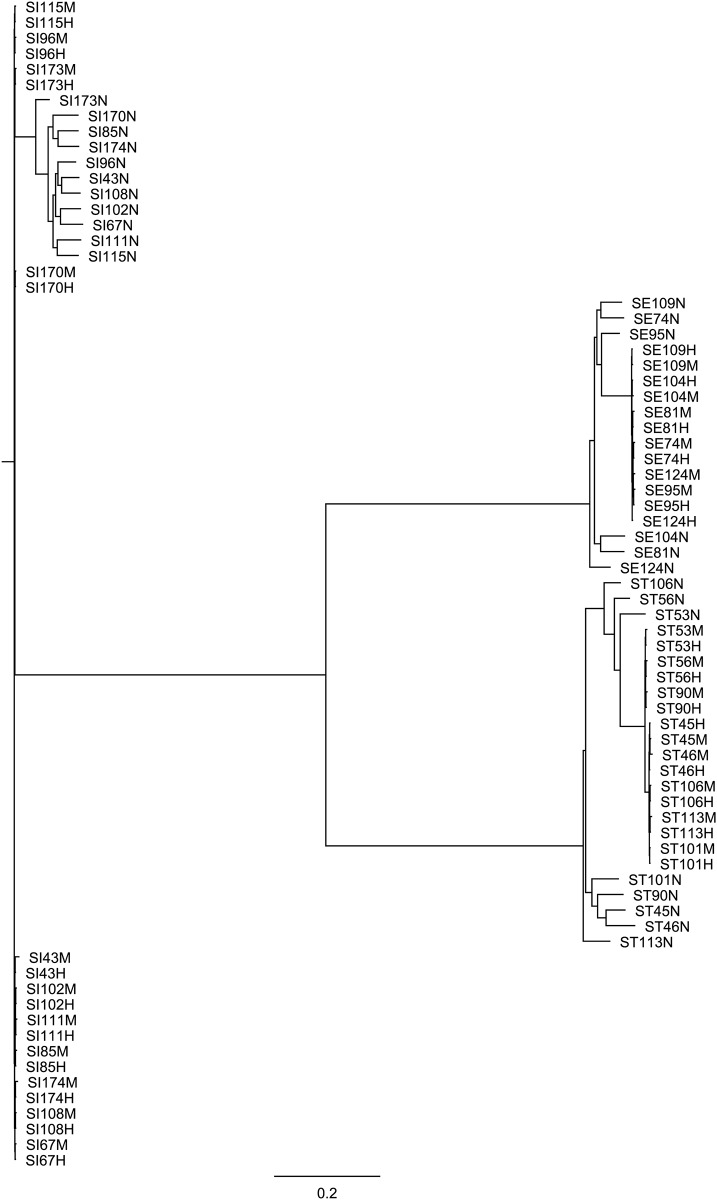
Whole-genome phylogenetic trees of *Salmonella* isolates based on their hybrid (H), MinION (N), and MiSeq (M) assemblies.

As shown in Fig 3, there was a greater distance from the MinION assembly clade to the other two clades relative to the distance between the MiSeq and Hybrid assembly clades. The lower read accuracy of MinION sequencing may have negatively affected the correct clustering of *Salmonella* isolates. The genetic relationships between the MiSeq and hybrid assemblies of each isolate were more concordant on the whole-genome phylogenetic tree, suggesting that the Unicycler hybrid assembly can be an effective strategy to generate genome assemblies that are both accurate and contiguous. Our overall finding was consistent with the work of González-Escalona et al. [55], which reported that the MinION assemblies of STEC had many errors against high-quality MiSeq and PacBio assemblies. Without polishing with the MiSeq short reads of STEC, the MinION assemblies were unable to be correctly placed onto the whole-genome phylogenetic tree.

*S*. Typhimurium LT2 ASM694v2 served as the reference genome for single nucleotide polymorphism (SNP) calling.

Because high-quality reference genomes are not always available, the MiSeq assemblies were considered as the “gold standard” to assess the accuracy of the MinION and hybrid assemblies in our study [55, 56]. Similarly, in the study of González-Escalona et al. [55] on the MinION assemblies of three *E*. *coli* O26:H11 strains, the MiSeq or HiSeq assemblies of 155 *E*. *coli* O26:H11 strains were considered as the “gold standard” for accurate genome sequence determination and SNP analyses. Taylor et al. [56] also used the MiSeq assembly as the reference genome for the *E*. *coli* O157:H7 isolate lacking a published reference genome when evaluating Nanopore sequencing technology for rapid phylogenetic inference. Although most MinION assemblies were more contiguous than the MiSeq assemblies, SNPs remained problematic in *de novo* assemblies generated from our MinION long reads. As indicated by our SNP analysis (S7 Table), 0.54–1.38 SNPs per kbp were detected in the MinION assemblies, which can, therefore, prevent accurate phylogenetic analysis due to errors in gene structure prediction. The Unicycler hybrid assembly reduced the number of SNPs to a lesser extent (<0.1 SNPs per kbp) with the combination of both short and long reads. All genome assemblies for the same serotype clustered together, with *S*. Typhimurium and Enteritidis having a closer genetic distance and being distinct from *S*. Indiana, implying that even the higher error rates of the MinION assemblies did not obscure serotype-level phylogenetic differences. Continued improvements in nanopore chemistry, as well as downstream base-calling and assembly, may mitigate the high numbers of SNPs. The potential application of MinION for epidemiological tracing during foodborne outbreaks remains to be validated utilizing more *Salmonella* isolates from diverse sources.

### Pan-genome analysis

**Fig 4:**
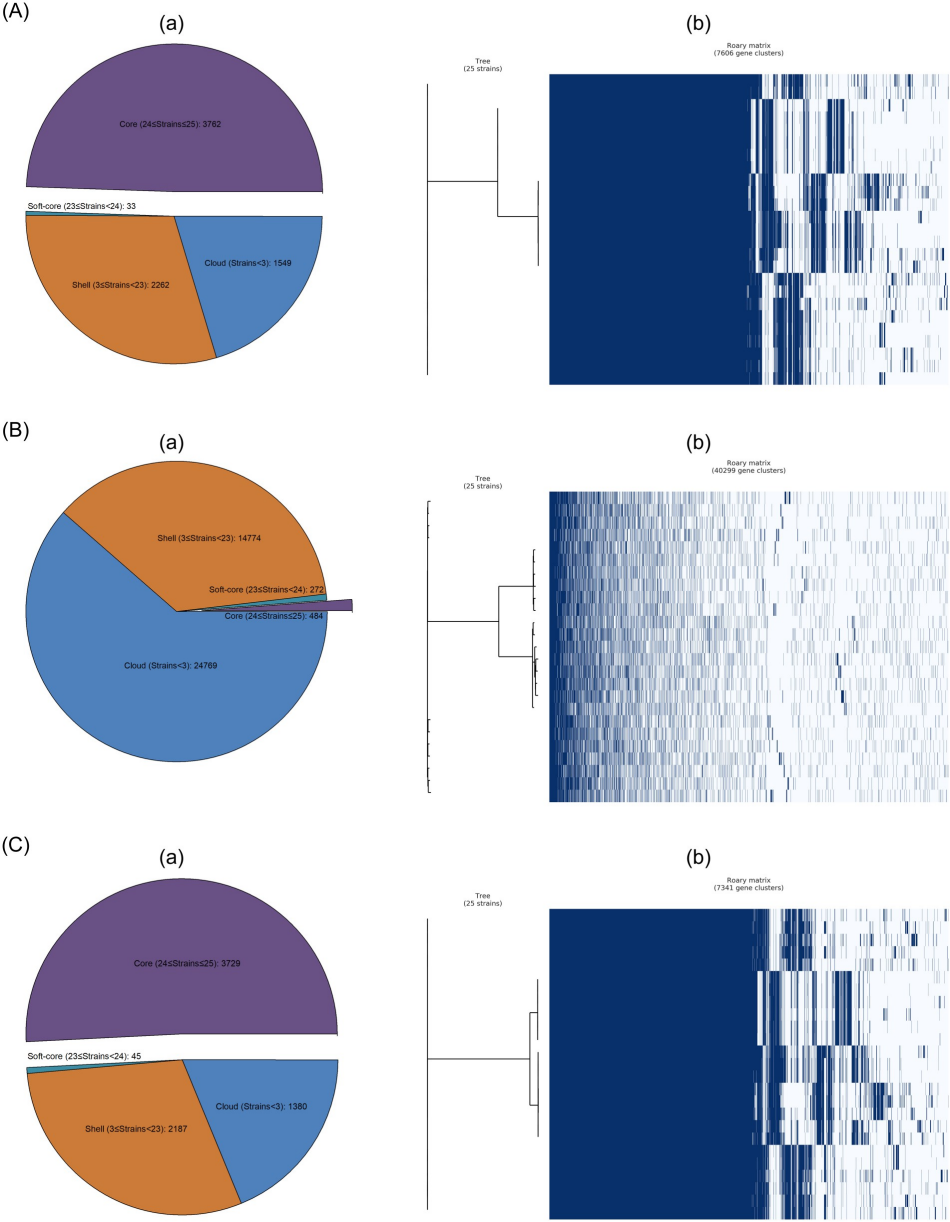
Pan-genome compositions (a), and core-genome phylogenetic trees compared to matrices with the presence and absence of core and accessory genes (b) of *Salmonella* isolates based on their hybrid (A), MinION (B), and MiSeq (C) assemblies.

The pan-genomes based on the genome annotations of the MiSeq and hybrid assemblies had similar numbers of core and accessory genes, as well as the matrices with the presence and absence of core and accessory genes, which were significantly different from the pan-genome of the MinION assemblies (Fig 4). The pan-genomes of the MiSeq and hybrid assemblies of 25 *Salmonella* isolates consisted of 7,341 genes with 3,729 core genes (50.8%) and 3,612 accessory genes (49.2%) and 7,606 genes with 3,762 core genes (49.5%) and 3,844 accessory genes (50.5%), respectively. In contrast, the total number of genes in the pan-genome of the MinION assemblies was significantly higher (40,299) with as many as 39,815 accessory genes (98.8%) and only 484 core genes (1.2%). Based on our core-genome phylogenetic analyses, the MinION assemblies on the core-genome phylogenetic tree were more genetically isolated from one another compared to the MiSeq and hybrid assemblies on their corresponding trees (trees are displayed on the left side of each matrix) (Fig 4), which was congruent with our pan-genome analyses.

This is likely due to the fact that MinION sequencing errors could introduce a stop codon and a start codon following an incorrectly introduced stop codon that truncated genes, which artificially increased gene counts in these assemblies [36]. High numbers of errors may, to some extent, interfere with high-quality genome annotations due to reduced inaccuracy in gene prediction to produce a large number of misannotated gene structures. Nonetheless, it should be noted that the hybrid assemblies possessed higher numbers of core and accessory genes than the MiSeq assemblies, suggesting the contribution of the MinION assemblies to these genes in the hybrid assemblies. The MinION assemblies may contain some genes that were unique to those particular genomes but were not annotated in the highly fragmented MiSeq assemblies due to the deficiencies that significantly lower the informational value of draft-quality genomes generated using short reads (S1–S3 Files). In a study by Jacobsen et al. [57], a comparative genomic analysis of 35 *Salmonella* genomes revealed that the addition of a fragmented genome can affect the size of the core and pan-genome proportionally more than the addition of a completed genome. Furthermore, since incomplete genomes may not always contain the full sequences for genes otherwise present, such truncated genes might erroneously be identified as novel gene families. Our finding in the current study thus further demonstrated that there was a tradeoff between assembly contiguity and annotation accuracy during the Unicycler hybrid assembly. Future research is necessary to specify the genes that were present and absent in the pan-genome of the MinION assemblies relative to the MiSeq and hybrid assemblies.

## Conclusions

We used MiSeq and MinION sequencing technologies, both individually and in combination, for the genomic analyses of 25 phenotypically multidrug-resistant isolates of *S*. Indiana, Typhimurium, and Enteritidis. A series of bioinformatic tools were used to translate raw sequence data into comprehensive genetic information. The MiSeq assemblies struggled to resolve genomic repeats and GC-rich regions, preventing assembly into complete genomes. Tradeoffs existed between the high contiguity of the MinION assemblies and their high numbers of errors, which was highlighted by our whole-genome phylogenetic and pan-genome analyses. Minimizing such errors of using Nanopore sequencing technology is thus warranted. Our study validated a framework to overcome these biases by combining the MinION long reads with the high-accuracy MiSeq short reads. The hybrid assembly typically generated assemblies that were both contiguous and that facilitated accurate annotations of complex genomic features. MinION significantly improved the genome-based high resolution for rapid detection and characterization of ARGs and virulence factors in *Salmonella*, although notable false negatives of tetracycline resistance were observed in some MinION assemblies. As nanopore chemistry and its relevant bioinformatic tools continue to evolve and improve, this long-read WGS technology, coupled with its increasing cost-effectiveness, is promising in providing a sufficient amount of data to complement the current WGS technologies for epidemiological inference and foodborne outbreak tracing.

## Supporting information

S1 Table Plasmid sizes of *Salmonella* isolates based on their hybrid assemblies. (DOCX)

S2 Table Plasmids of *Salmonella* isolates, as predicted based on their hybrid, MinION, and MiSeq assemblies. (DOCX)

S3 Table Antimicrobial resistance (AMR) phenotypes of *S*. Indiana isolates, and their corresponding AMR genotypes, as predicted based on their hybrid, MinION, and MiSeq assemblies. (DOCX)

S4 Table Antimicrobial resistance (AMR) phenotypes of *S*. Typhimurium isolates, and their corresponding AMR genotypes, as predicted based on their hybrid, MinION, and MiSeq assemblies. (DOCX)

S5 Table Antimicrobial resistance (AMR) phenotypes of *S*. Enteritidis isolates, and their corresponding AMR genotypes, as predicted based on their hybrid, MinION, and MiSeq assemblies. (DOCX)

S6 Table *Salmonella* pathogenicity islands (SPIs) of *Salmonella* isolates, as predicted based on their hybrid, MinION, and MiSeq assemblies. (DOCX)

S7 Table Single nucleotide polymorphisms (SNPs) in the hybrid and MinION assemblies of *Salmonella* isolates, as aligned to their corresponding MiSeq assemblies and expressed as SNPs per kbp. (DOCX)

S1 File Presence and absence of core and accessory genes of *Salmonella* isolates, as predicted based on their hybrid assemblies. (CSV)

S2 File Presence and absence of core and accessory genes of *Salmonella* isolates, as predicted based on their MinION assemblies. (CSV)

S3 File Presence and absence of core and accessory genes of *Salmonella* isolates, as predicted based on their MiSeq assemblies. (CSV)

## References

[1] Allard MW, Strain E, Melka D, Bunning K, Musser SM, Brown EW, et al Practical value of food pathogen traceability through building a whole-genome sequencing network and database. J Clin Microbiol. 2016;54:1975–83. 10.1128/JCM.00081-16 27008877 PMC4963501

[2] Quainoo S, Coolen JP, van Hijum SA, Huynen MA, Melchers WJ, van Schaik W, et al Whole-genome sequencing of bacterial pathogens: the future of nosocomial outbreak analysis. Clin Microbiol Rev. 2017;30:1015–63. 10.1128/CMR.00016-17 28855266 PMC5608882

[3] Gilchrist CA, Turner SD, Riley MF, Petri WA, Hewlett EL. Whole-genome sequencing in outbreak analysis. Clin Microbiol Rev. 2015;28:541–563. 10.1128/CMR.00075-13 25876885 PMC4399107

[4] Utturkar SM, Klingeman DM, Land ML, Schadt CW, Doktycz MJ, Pelletier DA, et al Evaluation and validation of *de novo* and hybrid assembly techniques to derive high-quality genome sequences. Bioinform. 2014;30:2709–2716. 10.1093/bioinformatics/btu391 PMC4173024 24930142

[5] Ashton PM, Nair S, Dallman T, Rubino S, Rabsch W, Mwaigwisya S, et al MinION nanopore sequencing identifies the position and structure of a bacterial antibiotic resistance island. Nat Biotechnol. 2015;33:296 10.1038/nbt.3103 25485618

[6] Madoui MA, Engelen S, Cruaud C, Belser C, Bertrand L, Alberti A, et al Genome assembly using Nanopore-guided long and error-free DNA reads. BMC Genomics. 2015;16:327 10.1186/s12864-015-1519-z 25927464 PMC4460631

[7] Rang FJ, Kloosterman WP, de Ridder J. From squiggle to basepair: computational approaches for improving nanopore sequencing read accuracy. Genome Biol. 2018;19:90 10.1186/s13059-018-1462-9 30005597 PMC6045860

[8] Jain M, Olsen HE, Paten B, Akeson M. The Oxford Nanopore MinION: delivery of nanopore sequencing to the genomics community. Genome Biol. 2016;17:239 10.1186/s13059-016-1103-0 27887629 PMC5124260

[9] Wick RR, Judd LM, Gorrie CL, Holt KE. Unicycler: resolving bacterial genome assemblies from short and long sequencing reads. PLOS Comput Biol. 2017;13:e1005595 10.1371/journal.pcbi.1005595 28594827 PMC5481147

[10] Antipov D, Korobeyniko A, McLean JS, Pevzner PA. hybridSPAdes: an algorithm for hybrid assembly of short and long reads. Bioinform. 2016;32:1009–1015. 10.1093/bioinformatics/btv688 PMC4907386 26589280

[11] Liao YS, Chen BH, Hong YP, Teng RH, Wang YW, Liang SY, et al Emergence of multidrug-resistant *Salmonella enterica* serovar Goldcoast strains in Taiwan and international spread of the ST358 clone. Antimicrob Agents Chemother. 2019;63:e01122–19. 10.1128/AAC.01122-19 31383653 PMC6761502

[12] Viñes J, Cuscó A, Francino O. Hybrid assembly from a pathogenic methicillin-and multidrug-resistant *Staphylococcus pseudintermedius* strain isolated from a case of canine otitis in Spain. Microbiol Resour Announc. 2020;9. 10.1128/MRA.01121-19 PMC6940279 31896627

[13] Hernández-Fillor RE, Brilhante M, Espinosa I, Perreten V. Complete circular genome sequence of a multidrug-resistant *Escherichia coli* strain from Cuba obtained with Nanopore and Illumina hybrid assembly. Microbiol Resour Announc. 2019;8. 10.1128/MRA.01269-19 PMC6883113 31776226

[14] Braden CR. *Salmonella enterica* serotype Enteritidis and eggs: a national epidemic in the United States. Clin Infect Dis. 2006;43:512–517. 10.1086/505973 16838242

[15] Carrasco E, Morales-Rueda A, García-Gimeno RM. Cross-contamination and recontamination by *Salmonella* in foods: a review. Food Res Int. 2012;45:545–56.

[16] Su M, Satola SW, Read TD. Genome-based prediction of bacterial antibiotic resistance. J Clin Microbiol. 2019;57:e01405–18. 10.1128/JCM.01405-18 30381421 PMC6425178

[17] Clinical and Laboratory Standards Institute (CLSI). Performance Standards for Antimicrobial Susceptibility Testing. 26th ed. CLSI Supplement M100-S26. Wayne: Clinical and Laboratory Standards Institute; 2016.

[18] Bolger AM, Lohse M, Usadel B. Trimmomatic: a flexible trimmer for Illumina sequence data. Bioinform. 2014;30:2114–2120. 10.1093/bioinformatics/btu170 PMC4103590 24695404

[19] Bankevich A, Nurk S, Antipov D, Gurevich AA, Dvorkin M, Kulikov AS, et al SPAdes: a new genome assembly algorithm and its applications to single-cell sequencing. J Comput Biol. 2012;19:455–477. 10.1089/cmb.2012.0021 22506599 PMC3342519

[20] De Coster W, D’Hert S, Schultz DT, Cruts M, Van Broeckhoven C. NanoPack: visualizing and processing long-read sequencing data. Bioinform. 2018;34:2666–2669. 10.1093/bioinformatics/bty149 PMC6061794 29547981

[21] Vaser R, Sović I, Nagarajan N, Šikić M. Fast and accurate de novo genome assembly from long uncorrected reads. Genome Res. 2017;27:737–746. 10.1101/gr.214270.116 28100585 PMC5411768

[22] Langmead B, Salzberg SL. Fast gapped-read alignment with Bowtie 2. Nat Methods. 2012;9:357 10.1038/nmeth.1923 22388286 PMC3322381

[23] Li H, Handsaker B, Wysoker A, Fennell T, Ruan J, Homer N, et al The sequence alignment/map format and SAMtools. Bioinform. 2009;25:2078–2079. 10.1093/bioinformatics/btp352 PMC2723002 19505943

[24] Walker BJ, Abeel T, Shea T, Priest M, Abouelliel A, Sakthikumar S, et al Pilon: an integrated tool for comprehensive microbial variant detection and genome assembly improvement. PLOS ONE. 2014;9:e112963 10.1371/journal.pone.0112963 25409509 PMC4237348

[25] Wick RR, Schultz MB, Zobel J, Holt KE. Bandage: interactive visualization of *de novo* genome assemblies. Bioinform. 2015;31:3350–3352. 10.1093/bioinformatics/btv383 PMC4595904 26099265

[26] Carattoli A, Zankari E, Garcìa-Fernandez A, Larsen MV, Lund O, Villa L, et al PlasmidFinder and pMLST: *in silico* detection and typing of plasmids. Antimicrob Agents Chemother. 2014:AAC-02412. 10.1128/AAC.02412-14 PMC4068535 24777092

[27] Zankari E, Allesøe R, Joensen KG, Cavaco LM, Lund O, Aarestrup FM. PointFinder: a novel web tool for WGS-based detection of antimicrobial resistance associated with chromosomal point mutations in bacterial pathogens. J Antimicrob Agents. 2017;72:2764–2768. 10.1093/jac/dkx217 PMC5890747 29091202

[28] Chen L, Yang J, Yu J, Yao Z, Sun L, Shen Y, et al VFDB: a reference database for bacterial virulence factors. Nucleic Acids Res. 2005;33:D325–D328. 10.1093/nar/gki008 15608208 PMC539962

[29] Kaas RS, Leekitcharoenphon P, Aarestrup FM, Lund O. Solving the problem of comparing whole bacterial genomes across different sequencing platforms. PLOS One. 2014;9:e104984 10.1371/journal.pone.0104984 25110940 PMC4128722

[30] Seemann T. Prokka: rapid prokaryotic genome annotation. Bioinform. 2014;30: 2068–9. 10.1093/bioinformatics/btu153 24642063

[31] Page AJ, Cummins CA, Hunt M, Wong VK, Reuter S, Holden MT, et al Roary: rapid large-scale prokaryote pan genome analysis. Bioinform. 2015;31:3691–3693. 10.1093/bioinformatics/btv421 PMC4817141 26198102

[32] Treangen TJ, Ondov BD, Koren S, Phillippy AM. Rapid core-genome alignment and visualization for thousands of microbial genomes. bioRxiv [Preprint]. 2014 bioRxiv 007351. [posted 2014 Jul 22] https://www.biorxiv.org/content/10.1101/007351v1 10.1186/s13059-014-0524-x PMC4262987 25410596

[33] Arndt D, Grant JR, Marcu A, Sajed T, Pon A, Liang Y, et al PHASTER: a better, faster version of the PHAST phage search tool. Nucleic Acids Res. 2016;44:W16–W21. 10.1093/nar/gkw387 27141966 PMC4987931

[34] Couvin D, Bernheim A, Toffano-Nioche C, Touchon M, Michalik J, Néron B, et al CRISPRCasFinder, an update of CRISRFinder, includes a portable version, enhanced performance and integrates search for Cas proteins. Nucleic Acids Res. 2018;46:W246–W251. 10.1093/nar/gky425 29790974 PMC6030898

[35] Hsu CH, Li C, Hoffmann M, McDermott P, Abbott J, Ayers S, et al Comparative genomic analysis of virulence, antimicrobial resistance, and plasmid profiles of *Salmonella* Dublin isolated from sick cattle, retail beef, and humans in the United States. Microb Drug Resist. 2019;25:1238–1249. 10.1089/mdr.2019.0045 31149890 PMC11555760

[36] Goldstein S, Beka L, Graf J, Klassen JL. Evaluation of strategies for the assembly of diverse bacterial genomes using MinION long-read sequencing. BMC Genomics. 2019;20:23 10.1186/s12864-018-5381-7 30626323 PMC6325685

[37] Lan JH, Yin Y, Reed EF, Moua K, Thomas K, Zhang Q. Impact of three Illumina library construction methods on GC bias and HLA genotype calling. Hum Immunol. 2015;76:166–175. 10.1016/j.humimm.2014.12.016 25543015 PMC5089167

[38] Eaves DJ, Randall L, Gray DT, Buckley A, Woodward MJ, White AP, et al Prevalence of mutations within the quinolone resistance-determining region of *gyrA*, *gyrB*, *parC*, and *parE* and association with antibiotic resistance in quinolone-resistant *Salmonella enterica*. Antimicrob Agents Chemother. 2004;48:4012–4015. 10.1128/AAC.48.10.4012-4015.2004 15388468 PMC521866

[39] Hopkins KL, Day M, Threlfall EJ. Plasmid-mediated quinolone resistance in *Salmonella enterica*, United Kingdom. Emerg Infect Dis. 2008;14:340 10.3201/eid1402.070573 18258138 PMC2600194

[40] Ruiz J. Transferable mechanisms of quinolone resistance from 1998 onward. Clin Microbiol Rev. 2019;32:e00007–19. 10.1128/CMR.00007-19 31413045 PMC6730498

[41] Jacoby GA, Chow N, Waites KB. Prevalence of plasmid-mediated quinolone resistance. Antimicrob Agents Chemother. 2003;47:559–562. 10.1128/aac.47.2.559-562.2003 12543659 PMC151764

[42] Nordmann P, Poirel L. Emergence of plasmid-mediated resistance to quinolones in Enterobacteriaceae. J Antimicrob Chemother. 2005;56:463–469. 10.1093/jac/dki245 16020539

[43] Wu W, Wang H, Lu J, Wu J, Chen M, Xu Y, et al Genetic diversity of *Salmonella enteric* serovar Typhi and Paratyphi in Shenzhen, China from 2002 through 2007. BMC Microbiol. 2010;10:32 10.1186/1471-2180-10-32 20113512 PMC2824697

[44] Nguyen F, Starosta AL, Arenz S, Sohmen D, Dönhöfer A, Wilson DN. Tetracycline antibiotics and resistance mechanisms. Biol Chem. 2014;395:559–575. 10.1515/hsz-2013-0292 24497223

[45] Feldgarden M, Brover V, Haft DH, Prasad AB, Slotta DJ, Tolstoy I, et al Validating the AMRFinder tool and resistance gene database by using antimicrobial resistance genotype-phenotype correlations in a collection of isolates. Antimicrob Agents Chemother. 2019;63:e00483–19. 10.1128/AAC.00483-19 31427293 PMC6811410

[46] Tyson GH, Li C, Ayers S, McDermott PF, Zhao S. Using whole-genome sequencing to determine appropriate streptomycin epidemiological cutoffs for *Salmonella* and *Escherichia coli*. FEMS Microbiol Lett. 2016; 363:fnw009 10.1093/femsle/fnw009 26781915 PMC11555754

[47] Tyson GH, Zhao S, Li C, Ayers S, Sabo JL, Lam C, et al Establishing genotypic cutoff values to measure antimicrobial resistance in *Salmonella*. Antimicrob Agents Chemother. 2017;61:e02140–16. 10.1128/AAC.02140-16 27993845 PMC5328538

[48] Partridge SR, Kwong SM, Firth N, Jensen SO. Mobile genetic elements associated with antimicrobial resistance. Clin Microbiol Rev. 2018;31:e00088–17. 10.1128/CMR.00088-17 30068738 PMC6148190

[49] van Belkum A, Soriaga LB, LaFave MC, Akella S, Veyrieras JB, Barbu EM, et al Phylogenetic distribution of CRISPR-Cas systems in antibiotic-resistant *Pseudomonas aeruginosa*. MBio. 2015;6:e01796–15. 10.1128/mBio.01796-15 26604259 PMC4669384

[50] DiMarzio M, Shariat N, Kariyawasam S, Barrangou R, Dudley EG. Antibiotic resistance in *Salmonella enterica* serovar Typhimurium associates with CRISPR sequence type. Antimicrob Agents Chemother. 2013;57:4282–4289. 10.1128/AAC.00913-13 23796925 PMC3754329

[51] Bearson BL, Allen HK, Brunelle BW, Lee IS, Casjens SR, Stanton TB. The agricultural antibiotic carbadox induces phage-mediated gene transfer in *Salmonella*. Front Microbiol. 2014;5:52 10.3389/fmicb.2014.00052 24575089 PMC3920066

[52] Bearson BL, Brunelle BW. Fluoroquinolone induction of phage-mediated gene transfer in multidrug-resistant *Salmonella*. Int J Antimicrob Agents. 2015;46:201–204. 10.1016/j.ijantimicag.2015.04.008 26078016

[53] Cao MD, Ganesamoorthy D, Elliott AG, Zhang H, Cooper MA, Coin LJ. Streaming algorithms for identification pathogens and antibiotic resistance potential from real-time MinION^™^ sequencing. Gigascience. 2016;5:s13742–016. 10.1186/s13742-016-0137-2 PMC4960868 27457073

[54] Břinda K, Callendrello A, Cowley L, Charalampous T, Lee RS, MacFadden DR, et al. Lineage calling can identify antibiotic resistant clones within minutes. bioRxiv [Preprint]. 2018 bioRxiv 403204. [posted 2019 Aug 7] https://www.biorxiv.org/content/10.1101/403204v1

[55] González-Escalona N, Allard MA, Brown EW, Sharma S, Hoffmann M. Nanopore sequencing for fast determination of plasmids, phages, virulence markers, and antimicrobial resistance genes in Shiga toxin-producing *Escherichia coli*. PLOS ONE. 2019;14:e0220494 10.1371/journal.pone.0220494 31361781 PMC6667211

[56] Taylor TL, Volkening JD, DeJesus E, Simmons M, Dimitrov KM, Tillman GE, et al Rapid, multiplexed, whole genome and plasmid sequencing of foodborne pathogens using long-read nanopore technology. Sci Rep. 2019;9:1–1. 31704961 10.1038/s41598-019-52424-x PMC6841976

[57] Jacobsen A, Hendriksen RS, Aaresturp FM, Ussery DW, Friis C. The *Salmonella enterica* pan-genome. Microb Ecol. 2011;62:487 10.1007/s00248-011-9880-1 21643699 PMC3175032

