# Supplementary material for: Genomic analyses of multidrug-resistant Salmonella Indiana, Typhimurium, and Enteritidis isolates using MinION and MiSeq sequencing technologies
Source: PLoS One. 2020 Jul 2;15(7):e0235641. doi: 10.1371/journal.pone.0235641 (PMC7332006; doi:10.1371/journal.pone.0235641)
Supplement: S1 Table — (DOCX) [file pone.0235641.s001.docx]

**S2 Table. Plasmid sizes of *Salmonella* isolates based on their hybrid assemblies.**

| Serotype | Isolate ID | Plasmid size (bp) |
| --- | --- | --- |
| Indiana | 43 | N.A.^a^ |
|  | 67 | 255,307 (1.08×)^b^ |
|  | 85 | 239,058 (0.96×), 4,234 (11.47×), 3,372(16.55×) |
|  | 96 | 190,808 (0.92×) |
|  | 102 | 199,036 (0.73×), 34,285 (2.92×), 4,677 (44.47×), 4,234 (27.65×), 2,677 (79.15×) |
|  | 108 | 145,548 (0.47×) |
|  | 111 | 30,641 (14.41×), 3,916 (75.16×), 3,373 (65.80×), 2,986 (35.64×) |
|  | 115 | 191,432 (0.42×) |
|  | 170 | 214,857 (0.41×) |
|  | 173 | 87,883 (1.02×), 29,164 (0.34×) |
|  | 174 | N.A. |
| Typhimurium | 45 | 260,432 (0.79×), 4,074 (21.17×) |
|  | 46 | 260,432 (0.85×), 4,074 (16.33×) |
|  | 53 | 115,084 (0.32×), 11,246 (6.83×) |
|  | 56 | 137,774 (0.82×), 108,574 (0.72×), 6,062 (19.45×), 4,715 (39.83×), 4,073 (18.90×) |
|  | 90 | 150,326 (1.05×), 110,163 (0.82×), 6,062 (10.50×), 4,715 (58.85×) |
|  | 101 | 181,971 (0.54×), 4,593 (18.76×) |
|  | 106 | 230,228 (0.77×), 2,708 (60.68×) |
|  | 113 | 147,578 (0.85×) |
| Enteritidis | 74 | 115,154 (4.23×), 64,336 (0.72×) |
|  | 81 | 59,372 (0.71×) |
|  | 95 | 90,315 (3.40×), 59,371 (0.95×), 7,264 (22.69×), 3,005 (0.40×) |
|  | 104 | 106,652 (3.66×), 59,372 (0.80×), 3,374 (54.46×) |
|  | 109 | 90,315 (2.43×), 59,372 (0.60×) |
|  | 124 | 108,447 (4.04×), 59,371 (0.93×) |

^a^N.A., not applicable due to no plasmids detected.

^b^The numbers in the parentheses indicate the depths of the plasmids.
