# Supplementary material for: Genomic analyses of multidrug-resistant Salmonella Indiana, Typhimurium, and Enteritidis isolates using MinION and MiSeq sequencing technologies
Source: PLoS One. 2020 Jul 2;15(7):e0235641. doi: 10.1371/journal.pone.0235641 (PMC7332006; doi:10.1371/journal.pone.0235641)
Supplement: S2 Table — (DOCX) [file pone.0235641.s002.docx]

**S3 Table. Plasmids of *Salmonella* isolates, as predicted based on their hybrid, MinION, and MiSeq assemblies.**

| Serotype | Isolate ID | Plasmid | | |
| --- | --- | --- | --- | --- |
|  |  | Hybrid | MinION | MiSeq |
| Indiana | 43 | IncHI2, IncHI2A, IncQ1 | IncHI2, IncHI2A, IncQ1 | IncHI2, IncHI2A, IncQ1 |
|  | 67 | IncHI2, IncHI2A, IncN | IncHI2, IncHI2A, IncN | IncHI2, IncHI2A, IncN |
|  | 85 | IncHI2, IncHI2A, IncN | IncHI2, IncHI2A, IncN | IncHI2, IncHI2A, IncN |
|  | 96 | IncHI2, IncHI2A, IncN | IncHI2, IncHI2A, IncN | IncHI2, IncHI2A, IncN |
|  | 102 | IncHI2, IncHI2A, IncN, IncX1 | IncHI2, IncHI2A, IncN | IncHI2, IncHI2A, IncN, IncX1 |
|  | 108 | IncFIA, IncFIB(AP001918), IncFII | IncFIA, IncFIB(AP001918), IncFII | IncFIA, IncFIB(AP001918), IncFII |
|  | 111 | IncQ1, IncX1 | IncQ1, IncX1 | IncQ1, IncX1 |
|  | 115 | IncHI2, IncHI2A, IncN | IncHI2, IncHI2A, IncN | IncHI2, IncHI2A, IncN |
|  | 170 | IncHI2, IncHI2A, IncN | IncHI2, IncHI2A, IncN | IncHI2, IncHI2A, IncN |
|  | 173 | IncI1, IncX1 | IncI1, IncX1 | IncI1, IncX1 |
|  | 174 | N.D. | N.D. | N.D. |
| Typhimurium | 45 | IncFII, IncHI2, IncHI2A, IncQ1 | IncFII, IncHI2, IncHI2A, IncQ1 | IncFII, IncHI2, IncHI2A, IncQ1 |
|  | 46 | IncFII, IncHI2, IncHI2A, IncQ1 | IncFII, IncHI2, IncHI2A, IncQ1 | IncFII, IncHI2, IncHI2A, IncQ1 |
|  | 53 | IncFIB(AP001918), IncFIB(S), IncFII(S), IncI1, IncX1 | IncFIB(AP001918), IncFIB(S), IncFII(S), IncI1, IncX1 | IncFIB(AP001918), IncFIB(S), IncFII(S), IncI1, IncX1 |
|  | 56 | IncA/C2, IncFIB(AP001918), IncFIB(S), IncFII(S) | IncA/C2, IncFIB(AP001918), IncFIB(S), IncFII(S) | IncA/C2, IncFIB(AP001918), IncFIB(S), IncFII(S) |
|  | 90 | IncFIA, IncFIB(AP001918), IncFIB(S), IncFII, IncFII(S), IncQ1 | IncFIA, IncFIB(AP001918), IncFIB(S), IncFII, IncFII(S), IncQ1 | IncFIA, IncFIB(AP001918), IncFIB(S), IncFII, IncFII(S), IncQ1 |
|  | 101 | IncA/C2 | IncA/C2 | IncA/C2 |
|  | 106 | IncHI2 , IncHI2A | IncHI2 , IncHI2A | IncHI2 , IncHI2A |
|  | 113 | IncA/C2 | IncA/C2 | IncA/C2 |
| Enteritidis | 74 | IncFIB(S), IncFII(S), IncFII(pHN7A8), IncN, IncX1 | IncFIB(S), IncFII(S), IncFII(pHN7A8), IncN, IncX1 | IncFIB(S), IncFII(S), IncFII(pHN7A8), IncN, IncX1 |
|  | 81 | IncFIB(S), IncFII(S) | IncFIB(S), IncFII(S) | IncFIB(S), IncFII(S) |
|  | 95 | IncFIB(S), IncFII(S), IncFII(pHN7A8), IncQ1), IncX1 | IncFIB(S), IncFII(S), IncFII(pHN7A8), IncQ1), IncX1 | IncFIB(S), IncFII(S), IncFII(pHN7A8), IncQ1), IncX1 |
|  | 104 | IncFIB(S), IncFII(S), IncFII(pHN7A8), IncX1 | IncFIB(S), IncFII(S), IncFII(pHN7A8), IncX1 | IncFIB(S), IncFII(S), IncFII(pHN7A8), IncX1 |
|  | 109 | IncFIB(S), IncFII(S), IncFII(pHN7A8), IncX1 | IncFIB(S), IncFII(S), IncFII(pHN7A8), IncX1 | IncFIB(S), IncFII(S), IncFII(pHN7A8), IncX1 |
|  | 124 | IncFIB(S), IncFII(S), IncFII(pHN7A8), IncX1 | IncFIB(S), IncFII(S), IncFII(pHN7A8), IncX1 | IncFIB(S), IncFII(S), IncFII(pHN7A8), IncX1 |
