# Supplementary material for: Genomic analyses of multidrug-resistant Salmonella Indiana, Typhimurium, and Enteritidis isolates using MinION and MiSeq sequencing technologies
Source: PLoS One. 2020 Jul 2;15(7):e0235641. doi: 10.1371/journal.pone.0235641 (PMC7332006; doi:10.1371/journal.pone.0235641)
Supplement: S5 Table — (DOCX) [file pone.0235641.s005.docx]

**S6 Table. Antimicrobial resistance (AMR) phenotypes of *S.* Enteritidis isolates, and their corresponding AMR genotypes, as predicted based on their hybrid, MinION, and MiSeq assemblies.^a^**

| Isolate | | | | 74 | | 81 | | 95 | | 104 | | 109 | | 124 | |
| --- | --- | --- | --- | --- | --- | --- | --- | --- | --- | --- | --- | --- | --- | --- | --- |
| ARGs | *β*-lactam | Phenotype | AMP^b^ |  |  |  |  |  |  |  |  |  |  |  |  |
|  |  |  | AMC |  |  |  |  |  |  |  |  |  |  |  |  |
|  |  |  | EFT |  |  |  |  |  |  |  |  |  |  |  |  |
|  |  |  | CEP |  |  |  |  |  |  |  |  |  |  |  |  |
|  |  |  | CRO |  |  |  |  |  |  |  |  |  |  |  |  |
|  |  |  | FOX |  |  |  |  |  |  |  |  |  |  |  |  |
|  |  | Genotype | *blaCTX-M* |  |  |  |  |  |  |  |  |  |  |  |  |
|  |  |  | *blaCTX-M-55* |  |  |  |  |  |  |  |  |  |  |  |  |
|  |  |  | *blaTEM* |  |  |  |  |  |  |  |  |  |  |  |  |
|  |  |  | *blaTEM-1* |  |  |  |  |  |  |  |  |  |  |  |  |
|  | Phenicol | Phenotype | CHL |  |  |  |  |  |  |  |  |  |  |  |  |
|  |  | Genotype | *floR* |  |  |  |  |  |  |  |  |  |  |  |  |
|  | Phenicol/Quinolone | Genotype | *oqxA* |  |  |  |  |  |  |  |  |  |  |  |  |
|  |  |  | *oqxA2* |  |  |  |  |  |  |  |  |  |  |  |  |
|  |  |  | *oqxB* |  |  |  |  |  |  |  |  |  |  |  |  |
|  | Quinolone | Phenotype | NAL |  |  |  |  |  |  |  |  |  |  |  |  |
|  |  |  | CIP |  |  |  |  |  |  |  |  |  |  |  |  |
|  |  |  | LVX |  |  |  |  |  |  |  |  |  |  |  |  |
|  |  |  | OFX |  |  |  |  |  |  |  |  |  |  |  |  |
|  | Aminoglycoside | Phenotype | AMK |  |  |  |  |  |  |  |  |  |  |  |  |
|  |  |  | GEN |  |  |  |  |  |  |  |  |  |  |  |  |
|  |  |  | KAN |  |  |  |  |  |  |  |  |  |  |  |  |
|  |  |  | STR |  |  |  |  |  |  |  |  |  |  |  |  |
|  |  | Genotype | *aac(3)-IV* |  |  |  |  |  |  |  |  |  |  |  |  |
|  |  |  | *aac(3)-IVa* |  |  |  |  |  |  |  |  |  |  |  |  |
|  |  |  | *aph(3'')-Ib* |  |  |  |  |  |  |  |  |  |  |  |  |
|  |  |  | *aph(3')-IIa* |  |  |  |  |  |  |  |  |  |  |  |  |
|  |  |  | *aph(6)-Id* |  |  |  |  |  |  |  |  |  |  |  |  |
|  | Tetracycline | Phenotype | TET |  |  |  |  |  |  |  |  |  |  |  |  |
|  |  | Genotype | *tet(A)* |  |  |  |  |  |  |  |  |  |  |  |  |
|  | Sulfonamide | Phenotype | SUL |  |  |  |  |  |  |  |  |  |  |  |  |
|  |  | Genotype | *sul2* |  |  |  |  |  |  |  |  |  |  |  |  |
|  | Trimethoprim | Phenotype | SXT |  |  |  |  |  |  |  |  |  |  |  |  |
|  | Bleomycin | Genotype | *ble* |  |  |  |  |  |  |  |  |  |  |  |  |
|  | Fosfomycin | Genotype | *fosA* |  |  |  |  |  |  |  |  |  |  |  |  |
|  |  |  | *fosA3* |  |  |  |  |  |  |  |  |  |  |  |  |
| Point mutations | Quinolone | Genotype | *gyrA* |  |  |  |  |  |  |  |  |  |  |  |  |
|  |  |  | *parC* |  |  |  |  |  |  |  |  |  |  |  |  |
|  | Colistin | Genotype | *pmrB* |  |  |  |  |  |  |  |  |  |  |  |  |

^a^Green, phenotypically positive; dark orange, genotypically positive as predicted based on the hybrid assemblies; cross, genotypically positive as predicted based on the MinION assemblies; light orange, genotypically positive as predicted based on the MiSeq assemblies.

^b^AMP, ampicillin; AMC, amoxicillin/clavulanic acid; EFT, ceftiofur; CEP, cefalexin; CRO, ceftriaxone; FOX, cefoxitin; CHL, chloramphenicol; NAL, nalidixic acid; CIP, ciprofloxacin; LVX, levofloxacin; OFX, ofloxacin; AMK, amikacin; GEN, gentamicin; KAN, kanamycin; STR, streptomycin; TET, tetracycline; SUL, sulfafurazole; SXT, trimethoprim/sulfamethoxazole.
