# Supplementary material for: Genomic analyses of multidrug-resistant Salmonella Indiana, Typhimurium, and Enteritidis isolates using MinION and MiSeq sequencing technologies
Source: PLoS One. 2020 Jul 2;15(7):e0235641. doi: 10.1371/journal.pone.0235641 (PMC7332006; doi:10.1371/journal.pone.0235641)
Supplement: S6 Table — (DOCX) [file pone.0235641.s006.docx]

**S7 Table. *Salmonella* pathogenicity islands (SPIs) of *Salmonella* isolates, as predicted based on their hybrid, MinION, and MiSeq assemblies.**

| Serotype | Isolate ID | SPIs | | |
| --- | --- | --- | --- | --- |
|  |  | Hybrid | MinION | MiSeq |
| Indiana | 43 | C63PI, SPI-3, SPI-4, SPI-5 | C63PI, SPI-3, SPI-4, SPI-5 | C63PI, SPI-1, SPI-3, SPI-4, SPI-5 |
|  | 67 | C63PI, SPI-2, SPI-3, SPI-4, SPI-5 | C63PI, SPI-2, SPI-3, SPI-4, SPI-5 | C63PI, SPI-2, SPI-3, SPI-4, SPI-5 |
|  | 85 | C63PI, SPI-2, SPI-3, SPI-4, SPI-5 | C63PI, SPI-2, SPI-3, SPI-4, SPI-5 | C63PI, SPI-2, SPI-3, SPI-5 |
|  | 96 | C63PI, SPI-2, SPI-3, SPI-4, SPI-5 | C63PI, SPI-2, SPI-3, SPI-4, SPI-5 | C63PI, SPI-1, SPI-2, SPI-4, SPI-5 |
|  | 102 | C63PI, SPI-1, SPI-2, SPI-3, SPI-4, SPI-5 | C63PI, SPI-1, SPI-2, SPI-3, SPI-4, SPI-5 | C63PI, SPI-1, SPI-2, SPI-3, SPI-4, SPI-5 |
|  | 108 | C63PI, SPI-2, SPI-3, SPI-4, SPI-5 | C63PI, SPI-1, SPI-2, SPI-3, SPI-4, SPI-5 | C63PI, SPI-2, SPI-3, SPI-4, SPI-5 |
|  | 111 | C63PI, SPI-2, SPI-3, SPI-5 | C63PI, SPI-2, SPI-3, SPI-5 | C63PI, SPI-1, SPI-2, SPI-3, SPI-5 |
|  | 115 | C63PI, SPI-2, SPI-3, SPI-4, SPI-5 | C63PI, SPI-2, SPI-3, SPI-4, SPI-5 | C63PI, SPI-1, SPI-2, SPI-3, SPI-4, SPI-5 |
|  | 170 | C63PI, SPI-2, SPI-3, SPI-4, SPI-5 | C63PI, SPI-1, SPI-2, SPI-3, SPI-4, SPI-5 | C63PI, SPI-1, SPI-2, SPI-3, SPI-4, SPI-5 |
|  | 173 | C63PI, SPI-2, SPI-4, SPI-5 | C63PI, SPI-2, SPI-4, SPI-5 | C63PI, SPI-2, SPI-4, SPI-5 |
|  | 174 | C63PI, SPI-2, SPI-3, SPI-4, SPI-5 | C63PI, SPI-1, SPI-2, SPI-3, SPI-4, SPI-5 | C63PI, SPI-1, SPI-2, SPI-3, SPI-4, SPI-5 |
| Typhimurium | 45 | C63PI, SPI-1, SPI-2, SPI-3, SPI-4, SPI-5, SPI-13, SPI-13, SPI-13, SPI-14, SPI-14 | C63PI, C63PI, SPI-2, SPI-3, SPI-4, SPI-5, SPI-13, SPI-13, SPI-13, SPI-14, SPI-14 | C63PI, SPI-1, SPI-2, SPI-3, SPI-5, SPI-13, SPI-13, SPI-13, SPI-14, SPI-14 |
|  | 46 | C63PI, SPI-1, SPI-2, SPI-3, SPI-4, SPI-5, SPI-13, SPI-13, SPI-13, SPI-14, SPI-14 | C63PI, C63PI, SPI-2, SPI-3, SPI-4, SPI-5, SPI-13, SPI-13, SPI-13, SPI-14, SPI-14 | C63PI, SPI-1, SPI-2, SPI-3, SPI-5, SPI-13, SPI-13, SPI-13, SPI-14, SPI-14 |
|  | 53 | C63PI, SPI-1, SPI-2, SPI-3, SPI-4, SPI-5, SPI-13, SPI-13, SPI-13, SPI-14, SPI-14 | C63PI, C63PI, SPI-2, SPI-3, SPI-4, SPI-5, SPI-13, SPI-13, SPI-13, SPI-14, SPI-14 | C63PI, SPI-1, SPI-2, SPI-3, SPI-5, SPI-13, SPI-13, SPI-13, SPI-14, SPI-14 |
|  | 56 | C63PI, SPI-1, SPI-2, SPI-3, SPI-4, SPI-5, SPI-13, SPI-13, SPI-13, SPI-14, SPI-14 | C63PI, C63PI, SPI-2, SPI-3, SPI-4, SPI-5, SPI-13, SPI-13, SPI-13, SPI-14, SPI-14 | C63PI, SPI-1, SPI-2, SPI-3, SPI-5, SPI-13, SPI-13, SPI-13, SPI-14, SPI-14 |
|  | 90 | C63PI, SPI-1, SPI-2, SPI-3, SPI-4, SPI-5, SPI-13, SPI-13, SPI-13, SPI-14, SPI-14 | C63PI, C63PI, SPI-2, SPI-3, SPI-4, SPI-5, SPI-13, SPI-13, SPI-13, SPI-14, SPI-14 | C63PI, SPI-1, SPI-2, SPI-3, SPI-5, SPI-13, SPI-13, SPI-13, SPI-14, SPI-14 |
|  | 101 | C63PI, C63PI, SPI-1, SPI-2, SPI-3, SPI-4, SPI-5, SPI-13, SPI-13, SPI-13, SPI-14, SPI-14 | C63PI, C63PI, SPI-1, SPI-2, SPI-3, SPI-4, SPI-5, SPI-13, SPI-13, SPI-13, SPI-14, SPI-14 | C63PI, C63PI, SPI-1, SPI-2, SPI-3, SPI-5, SPI-13, SPI-13, SPI-13, SPI-14, SPI-14 |
|  | 106 | C63PI, SPI-1, SPI-2, SPI-3, SPI-4, SPI-5, SPI-13, SPI-13, SPI-13, SPI-14, SPI-14 | C63PI, C63PI, SPI-2, SPI-3, SPI-5, SPI-13, SPI-13, SPI-13, SPI-14, SPI-14 | C63PI, SPI-1, SPI-2, SPI-3, SPI-5, SPI-13, SPI-13, SPI-13, SPI-14, SPI-14 |
|  | 113 | C63PI, SPI-1, SPI-2, SPI-3, SPI-4, SPI-5, SPI-13, SPI-13, SPI-13, SPI-14, SPI-14 | C63PI, C63PI, SPI-1, SPI-2, SPI-3, SPI-4, SPI-5, SPI-13, SPI-13, SPI-13, SPI-14, SPI-14 | C63PI, SPI-1, SPI-2, SPI-3, SPI-5, SPI-13, SPI-13, SPI-13, SPI-14, SPI-14 |
| Enteritidis | 74 | C63PI, SPI-1, SPI-3, SPI-4, SPI-5, SPI-12, SPI-13, SPI-13, SPI-13, SPI-14, SPI-14 | C63PI, SPI-1, SPI-3, SPI-4, SPI-5, SPI-12, SPI-13, SPI-13, SPI-13, SPI-14, SPI-14 | C63PI, SPI-1, SPI-3, SPI-4, SPI-5, SPI-13, SPI-13, SPI-13, SPI-14, SPI-14 |
|  | 81 | C63PI, SPI-1, SPI-3, SPI-4, SPI-5, SPI-12, SPI-13, SPI-13, SPI-13, SPI-14, SPI-14 | C63PI, SPI-1, SPI-3, SPI-4, SPI-5, SPI-12, SPI-13, SPI-13, SPI-13, SPI-14, SPI-14 | C63PI, SPI-1, SPI-3, SPI-4, SPI-5, SPI-13, SPI-13, SPI-13, SPI-14, SPI-14 |
|  | 95 | C63PI, SPI-1, SPI-3, SPI-4, SPI-5, SPI-12, SPI-13, SPI-13, SPI-13, SPI-14, SPI-14 | C63PI, SPI-1, SPI-3, SPI-4, SPI-5, SPI-12, SPI-13, SPI-13, SPI-13, SPI-14, SPI-14 | C63PI, SPI-1, SPI-3, SPI-4, SPI-5, SPI-13, SPI-13, SPI-13, SPI-14, SPI-14 |
|  | 104 | C63PI, SPI-1, SPI-3, SPI-4, SPI-5, SPI-12, SPI-13, SPI-13, SPI-13, SPI-14, SPI-14 | C63PI, SPI-1, SPI-3, SPI-4, SPI-5, SPI-12, SPI-13, SPI-13, SPI-13, SPI-14, SPI-14 | C63PI, SPI-1, SPI-3, SPI-5, SPI-13, SPI-13, SPI-13, SPI-14, SPI-14 |
|  | 109 | C63PI, SPI-1, SPI-3, SPI-4, SPI-5, SPI-12, SPI-13, SPI-13, SPI-13, SPI-14, SPI-14 | C63PI, SPI-1, SPI-3, SPI-4, SPI-5, SPI-12, SPI-13, SPI-13, SPI-13, SPI-14, SPI-14 | C63PI, SPI-1, SPI-3, SPI-4, SPI-5, SPI-13, SPI-13, SPI-13, SPI-14, SPI-14 |
|  | 124 | C63PI, SPI-1, SPI-3, SPI-4, SPI-5, SPI-12, SPI-13, SPI-13, SPI-13, SPI-14, SPI-14 | C63PI, SPI-1, SPI-3, SPI-4, SPI-5, SPI-12, SPI-13, SPI-13, SPI-13, SPI-14, SPI-14 | C63PI, SPI-1, SPI-3, SPI-4, SPI-5, SPI-13, SPI-13, SPI-13, SPI-14, SPI-14 |
