# Supplementary material for: Genomic analyses of multidrug-resistant Salmonella Indiana, Typhimurium, and Enteritidis isolates using MinION and MiSeq sequencing technologies
Source: PLoS One. 2020 Jul 2;15(7):e0235641. doi: 10.1371/journal.pone.0235641 (PMC7332006; doi:10.1371/journal.pone.0235641)
Supplement: S7 Table — (DOCX) [file pone.0235641.s007.docx]

**S8 Table. Single nucleotide polymorphisms (SNPs) in the hybrid and MinION assemblies of *Salmonella* isolates, as aligned to their corresponding MiSeq assemblies and expressed as SNPs per kbp.**

| Serotype | Isolate ID | SNPs per kbp | |
| --- | --- | --- | --- |
|  |  | Hybrid | MinION |
| Indiana | 43 | 0.08 | 1.34 |
|  | 67 | 0.02 | 1.34 |
|  | 85 | 0.03 | 1.37 |
|  | 96 | 0.02 | 1.26 |
|  | 102 | 0.01 | 1.38 |
|  | 108 | 0.01 | 1.21 |
|  | 111 | 0.02 | 1.37 |
|  | 115 | 0.01 | 1.37 |
|  | 170 | 0.01 | 1.34 |
|  | 173 | 0.02 | 1.16 |
|  | 174 | 0.05 | 1.34 |
| Typhimurium | 45 | 0.01 | 1.08 |
|  | 46 | 0.04 | 1.13 |
|  | 53 | 0.02 | 0.65 |
|  | 56 | 0.02 | 0.54 |
|  | 90 | 0.02 | 1.00 |
|  | 101 | 0.00 | 0.84 |
|  | 106 | 0.01 | 0.57 |
|  | 113 | 0.03 | 0.89 |
| Enteritidis | 74 | 0.02 | 0.99 |
|  | 81 | 0.03 | 1.03 |
|  | 95 | 0.03 | 0.92 |
|  | 104 | 0.02 | 1.01 |
|  | 109 | 0.02 | 0.94 |
|  | 124 | 0.03 | 0.99 |
